# Littlewood–Paley Theory for Triangle Buildings

**DOI:** 10.1007/s12220-017-9856-6

**Published:** 2017-05-08

**Authors:** Tim Steger, Bartosz Trojan

**Affiliations:** 1 Matematica, Università degli Studi di Sassari, Via Piandanna 4, 07100 Sassari, Italy; 2 Wydział Matematyki, Politechnika Wrocławska, Wyb. Wyspiańskiego 27, 50-370 Wrocław, Poland

**Keywords:** Affine building, Littlewood–Paley theory, Square function, Maximal function, Multi-index filtration, Heisenberg group, p-adic numbers, Primary 22E35, 51E24, 60G42

## Abstract

For the natural two-parameter filtration $\left( {\mathcal {F}_\lambda }: {\lambda \in P}\right)$ on the boundary of a triangle building, we define a maximal function and a square function and show their boundedness on $L^p(\Omega _0)$ for $p \in (1, \infty )$. At the end, we consider $L^p(\Omega _0)$ boundedness of martingale transforms. If the building is of ${\text {GL}}(3, \mathbb {Q}_p)$, then $\Omega _0$ can be identified with *p*-adic Heisenberg group.

## Introduction

Let $(\Omega , \mathcal {F}, \pi )$ be a $\sigma$-finite measure space. A sequence of $\sigma$-algebras $(\mathcal {F}_n : n \in \mathbb {Z})$ is a filtration if $\mathcal {F}_n \subset \mathcal {F}_{n+1}$. Given *f* a locally integrable function on $\Omega$ by $\mathbb {E}[f | \mathcal {F}_n]$, we denote its conditional expectation value with respect to $\mathcal {F}_n$. Let $M^*$ and *S* denote, respectively, the maximal function and the square function defined by

$$\begin{aligned} M^* f = \sup _{n \in \mathbb {Z}} {|{f_n} |}, \end{aligned}$$

and

1.1 $$\begin{aligned} S f = \Big (\sum _{n \in \mathbb {Z}} {|{d_n f} |}^2 \Big )^{1/2}, \end{aligned}$$

where $d_n f = f_n - f_{n-1}$. The Hardy and Littlewood maximal estimate (see [8]) implies that

$$\begin{aligned} \pi \big (\big \{ M^* f> \lambda \big \}\big ) \le \lambda ^{-1} \int _{M^* f > \lambda } {|{f} |} {\, \mathrm d}\pi , \end{aligned}$$

from where it is easy to deduce that for $p \in (1, \infty ]$

$$\begin{aligned} {\left||M^* f \right||}_{L^p} \le \frac{p}{p-1} {\left||f \right||}_{L^p}. \end{aligned}$$

For the square function, if $p \in (1, \infty )$, then there is $C_p > 1$ such that

1.2 $$\begin{aligned} C_p^{-1} {\left||f \right||}_{L^p} \le {\left||S f \right||}_{L^p} \le C_p {\left||f \right||}_{L^p}. \end{aligned}$$

The inequality (1.2) goes back to Paley [12], and has been reproved in many ways, for example, [2–4, 7, 10]. Its main application is in proving the $L^p$-boundedness of martingale transforms (see [2]), that is, for operators of the form

$$\begin{aligned} T f = \sum _{n \in \mathbb {Z}} a_n d_n f \end{aligned}$$

where $(a_n : n \in \mathbb {Z})$ is a sequence of uniformly bounded functions such that $a_{n+1}$ is $\mathcal {F}_n$-measurable.

In 1975, Cairoli and Walsh (see [5]) have started to generalize the theory of martingales to two-parameter cases. Let us recall that a sequence of $\sigma$-fields $(\mathcal {F}_{n, m} : n, m \in \mathbb {Z})$ is a two-parameter filtration if

1.3 $$\begin{aligned} \mathcal {F}_{n+1, m} \subset \mathcal {F}_{n, m}, \qquad \text {and} \qquad \mathcal {F}_{n, m+1} \subset \mathcal {F}_{n, m}. \end{aligned}$$

Then $(f_{n, m} : n, m \in \mathbb {Z})$ is a two-parameter martingale if

1.4 $$\begin{aligned} \mathbb {E}[f_{n+1, m} | \mathcal {F}_{n, m}] = f_{n, m}, \qquad \text {and} \qquad \mathbb {E}[f_{n, m+1} | \mathcal {F}_{n, m}] = f_{n, m}. \end{aligned}$$

Observe that conditions (1.3) and (1.4) impose a structure only for comparable indices. In that generality, it is hard, if not impossible, to build the Littlewood–Paley theory. This lead to the introduction of other (smaller) classes of martingales (see [19, 20]). In particular, in [5], Cairoli and Walsh introduced the following condition 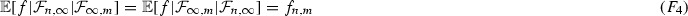 where

$$\begin{aligned} \mathcal {F}_{n, \infty } = \sigma \Big (\bigcup _{m \in \mathbb {Z}} \mathcal {F}_{n ,m} \Big ), \qquad \text {and} \qquad \mathcal {F}_{\infty , m} = \sigma \Big (\bigcup _{n \in \mathbb {Z}} \mathcal {F}_{n, m} \Big ). \end{aligned}$$

Under ($F_4$), the result obtained by Jensen, Marcinkiewicz, and Zygmund in [9] implies that the maximal function

1.5 $$\begin{aligned} M^* f = \sup _{n, m \in \mathbb {Z}} {|{f_{n, m}} |} \end{aligned}$$

is bounded on $L^p(\Omega )$ for $p \in (1, \infty ]$. In this context, the square function is defined by

1.6 $$\begin{aligned} S f = \Big (\sum _{n, m \in \mathbb {Z}} {|{d_{n, m} f} |}^2 \Big )^{1/2} \end{aligned}$$

where $d_{n, m}$ denote the double difference operator, i.e.

$$\begin{aligned} d_{n, m} f = f_{n, m} - f_{n-1, m} - f_{n, m-1} + f_{n-1, m-1}. \end{aligned}$$

In [11], it was observed by Metraux that the boundedness of *S* on $L^p(\Omega )$ for $p \in (1, \infty )$ is implied by the one parameter Littlewood–Paley theory. Also the concept of a martingale transform has a natural generalization, that is,

$$\begin{aligned} T f = \sum _{n, m \in \mathbb {Z}} a_{n, m} d_{n, m} f \end{aligned}$$

where $(a_{n, m} : n,m \in \mathbb {Z})$ is a sequence of uniformly bounded functions such that $a_{n+1, m+1}$ is $\mathcal {F}_{n, m}$-measurable.

In this article, we are interested in a case when the condition ($F_4$) is not satisfied. The simplest example may be obtained by considering the Heisenberg group together with the non-isotropic two parameter dilations

$$\begin{aligned} \delta _{s, t} (x, y, z) = (s x, t y, st z). \end{aligned}$$

Since in this setup the dyadic cubes do not posses the same properties as the Euclidean cubes, it is more convenient to work on the *p*-adic version of the Heisenberg group. We observe that this group can be identified with $\Omega _0$, a subset of a boundary of the building of ${\text {GL}}(3, \mathbb {Q}_p)$ consisting of the points opposite to a given $\omega _0$. The set $\Omega _0$ has a natural two-parameter filtration $(\mathcal {F}_{n, m} : n, m \in \mathbb {Z})$ (see Sect. 2 for details). The maximal function and the square function are defined by (1.5) and (1.6), respectively. The results we obtain are summarized in the following three theorems.

### Theorem A

For each $p \in (1, \infty ]$, there is $C_p > 0$ such that for all $f \in L^p\big (\Omega _0\big )$

$$\begin{aligned} {\left||M^*f \right||}_{L^p} \le C_p {\left||f \right||}_{L^p}. \end{aligned}$$

### Theorem B

For each $p \in (1, \infty )$, there is $C_p > 1$ such that for all $f \in L^p\big (\Omega _0\big )$

$$\begin{aligned} C_p^{-1} {\left||f \right||}_{L^p} \le {\left||S f \right||}_{L^p} \le C_p {\left||f \right||}_{L^p}. \end{aligned}$$

### Theorem C

If $(a_{n, m} : n, m \in \mathbb {Z})$ is a sequence of uniformly bounded functions such that $a_{n+1, m+1}$ is $\mathcal {F}_{n, m}$-measurable, then the martingale transform

$$\begin{aligned} T f = \sum _{n, m \in \mathbb {Z}} a_{n, m} d_{n, m} f \end{aligned}$$

is bounded on $L^p\big (\Omega _0\big )$, for all $p \in (1, \infty )$.

Let us briefly describe methods we use. First, we observe that instead of ($F_4$) the stochastic basis satisfies the remarkable identity (2.2). Based on it, we show that the following pointwise estimate holds

1.7 $$\begin{aligned} M^*({|{f} |}) \le C \big (L^* R^* L^* R^*({|{f} |}) + R^* L^* R^* L^* ({|{f} |}) \big ) \end{aligned}$$

proving the maximal theorem. Thanks to the two-parameter Khintchine’s inequality, to bound the square function *S*, it is enough to show Theorem C. To do so, we define a new square function $\mathcal {S}$ which has a nature similar to the square function used in the presence of ($F_4$). Then, we adapt the technique developed by Duoandikoetxea and Rubio de Francia in [6] (see Theorem 3). This implies $L^p$-boundedness of *S*. Since *S* does not preserve the $L^2$ norm, the lower bound requires an extra argument. Namely, we view the square function *S* as an operator with values in $L^p(\ell ^2)$ and take its dual. As a consequence of Theorem 3 and the identity (4.7), the latter is bounded on $L^p$.

Finally, let us comment on the behavior of the maximal function $M^*$ close to $L^1$. Based on the pointwise estimate (1.7), in view of [8], we conclude that $M^*$ is of weak-type for functions in the Orlicz space $L (\log L)^3$. To better understand the maximal function $M^*$, we investigate exact behavior close to $L^1$. This together with weighted estimates is the subject of the forthcoming paper. It is also interesting how to extend Theorems A, B and C to higher rank and other types of affine buildings.

### Notation

For two quantities $A>0$ and $B>0$, we say that $A \lesssim B$ ($A \gtrsim B$) if there exists an absolute constant $C>0$ such that $A\le CB$ ($A\ge CB$).

If $\lambda \in P$ we set ${|{\lambda } |} = \max \left\{ {|{\lambda _1} |}, {|{\lambda _2} |}\right\}$.

## Triangle Buildings

### Coxeter Complex

We recall basic facts about the $A_2$ root system and the $\tilde{A}_2$ Coxeter group. A general reference is [1]. Let $\mathfrak {a}$ be the hyperplane in $\mathbb {R}^3$ defined as

$$\begin{aligned} \mathfrak {a} = \{(x_1, x_2, x_3) \in \mathbb {R}^3: x_1 + x_2 + x_3 = 0 \}. \end{aligned}$$

We denote by $\{e_1, e_2, e_3\}$ the canonical orthonormal basis of $\mathbb {R}^3$ with respect to the standard scalar product ${\langle \cdot , \cdot \rangle }$. We set $\alpha _1 = e_2 - e_1$, $\alpha _2 = e_3 - e_2$, $\alpha _0 = e_3 - e_1$ and $I = \{0, 1, 2 \}$. The $A_2$ root system is defined by

$$\begin{aligned} \Phi = \{\pm \alpha _0, \pm \alpha _1, \pm \alpha _2\}. \end{aligned}$$

We choose the base $\{\alpha _1, \alpha _2\}$ of $\Phi$. The corresponding positive roots are $\Phi ^+ = \{\alpha _0, \alpha _1, \alpha _2\}$. Denote by $\{\lambda _1, \lambda _2\}$ the basis dual to $\{\alpha _1, \alpha _2\}$; its elements are called the *fundamental co-weights*. Their integer combinations, form the *co-weight lattice* *P*. As

**Fig. 1:**
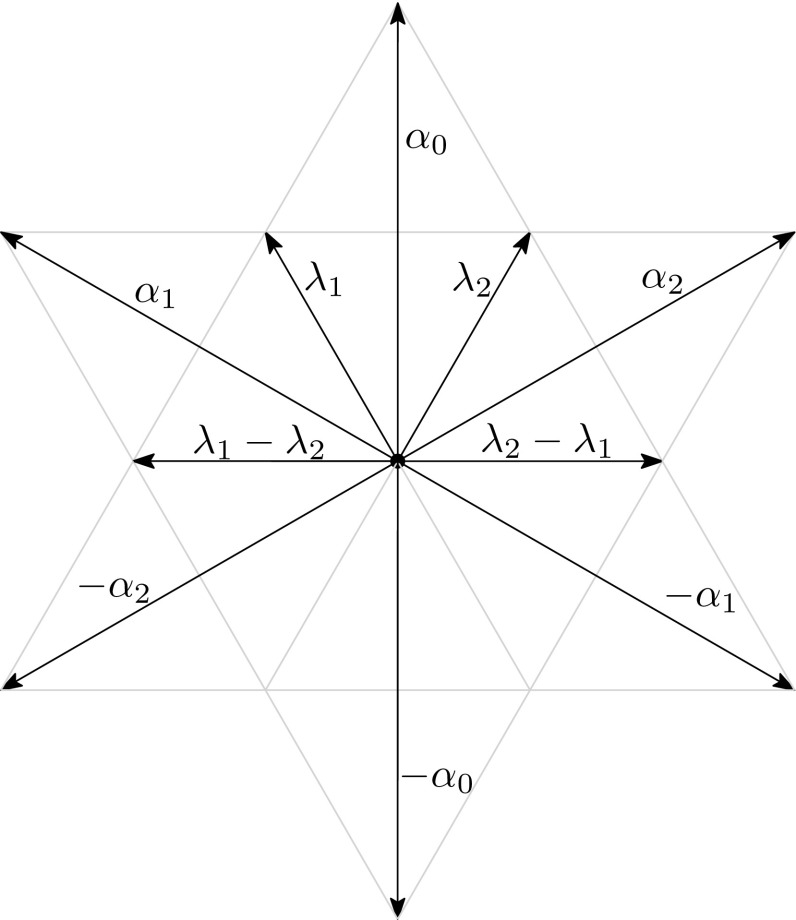
$$A_2$$ root system

in Fig. 1, we always draw $\lambda _1$ pointing up and to the left and $\lambda _2$ up and to the right. Likewise $\lambda _1-\lambda _2$ is drawn pointing directly left, while $\lambda _2-\lambda _1$ points directly right. Because ${\langle \lambda _1, \alpha _0\rangle }={\langle \lambda _2, \alpha _0\rangle }=1$, we see that for any $\lambda \in P$ the expression ${\langle \lambda , \alpha _0\rangle }$ represents the vertical level of $\lambda$. For $\lambda =i\lambda _1+j\lambda _2$, that level is $i+j$.

Let $\mathcal {H}$ be the family of affine hyperplanes, called *walls*,

$$\begin{aligned} H_{j; k}=\{x \in \mathfrak {a}: {\langle x, \alpha _j\rangle } = k\} \end{aligned}$$

where $j \in I$, $k \in \mathbb {Z}$. To each wall $H_{j; k}$, we associate $r_{j;k}$ the orthogonal reflection in $\mathfrak {a}$, i.e.

$$\begin{aligned} r_{j; k} (x) = x - \big ({\langle x, \alpha _j\rangle } - k\big ) \alpha _j. \end{aligned}$$

Set $r_1 = r_{1; 0}$, $r_2 = r_{2; 0}$ and $r_0 = r_{0; 1}$. The *finite Weyl group* $W_0$ is the subgroup of ${\text {GL}}(\mathfrak {a})$ generated by $r_1$ and $r_2$. The *affine Weyl group* *W* is the subgroup of ${\text {Aff}}(\mathfrak {a})$ generated by $r_0$, $r_1$ and $r_2$.

Let $\mathcal {C}$ be the family of open connected components of $\mathfrak {a} \setminus \bigcup _{H \in \mathcal {H}} H$. The elements of $\mathcal {C}$ are called *chambers*. By $C_0$, we denote the fundamental chamber, i.e.

$$\begin{aligned} C_0 = \{x \in \mathfrak {a}: {\langle x, \alpha _1\rangle }> 0, {\langle x, \alpha _2\rangle } > 0, {\langle x, \alpha _0\rangle } < 1\}. \end{aligned}$$

The group *W* acts simply transitively on $\mathcal {C}$. Moreover, $\overline{C_0}$ is a fundamental domain for the action of *W* on $\mathfrak {a}$ (see e.g. [1, VI, §1-3]). The vertices of $C_0$ are $\{0, \lambda _1, \lambda _2\}$. The set of all vertices of all $C\in \mathcal {C}$ is denoted by $V(\Sigma )$. Under the action of *W*, $V(\Sigma )$ is made up of three orbits, *W*(0), $W(\lambda _1)$, and $W(\lambda _2)$. Vertices in the same orbit are said to have the same *type*. Any chamber $C\in \mathcal {C}$ has one vertex in each orbit or in other words one vertex of each of the three types.

The family $\mathcal {C}$ may be regarded as a simplicial complex $\Sigma$ by taking as the simplexes all non-empty subsets of vertices of *C*, for all $C \in \mathcal {C}$. Two chambers *C* and $C^{\prime }$ are *i*-*adjacent* for $i \in I$ if $C = C'$ or if there is $w \in W$ such that $C=wC_0$ and $C^{\prime }=wr_iC_0$. Since $r_i^2=1$ this defines an equivalence relation.

The *fundamental sector* is defined by

$$\begin{aligned} \mathcal {S}_0 = \{x \in \mathfrak {a}: {\langle x, \alpha _1\rangle }> 0, {\langle x, \alpha _2\rangle } > 0\}. \end{aligned}$$

Given $\lambda \in P$ and $w \in W_0$ the set $\lambda + w \mathcal {S}_0$ is called a *sector* in $\Sigma$ with *base vertex* $\lambda$. The angle spanned by a sector at its base vertex is $\pi /3$.

### The Definition of Triangle Buildings

For the theory of affine buildings, we refer the reader to [13]. See also the first author’s expository paper [14], for an elementary introduction to the *p*-adics, and to precisely the sort of the buildings which this paper deals with.

A simplicial complex $\mathscr {X}$ is an $\tilde{A}_2$ *building*, or as we like to call it, a *triangle building*, if each of its vertices is assigned one of the three types, and if it contains a family of subcomplexes called *apartments* such that

1. Each apartment is type-isomorphic to $\Sigma$,
2. Any two simplexes of $\mathscr {X}$ lie in a common apartment,
3. For any two apartments, $\mathscr {A}$ and $\mathscr {A}^{\prime }$, having a chamber in common, there is a type-preserving isomorphism $\psi : \mathscr {A} \rightarrow \mathscr {A}^{\prime }$ fixing $\mathscr {A} \cap \mathscr {A}^{\prime }$ pointwise.

We assume also that the system of apartments is *complete*, meaning that any subcomplex of $\mathscr {X}$ type-isomorphic to $\Sigma$ is an apartment. A simplex *C* is a *chamber* in $\mathscr {X}$ if it is a chamber for some apartment. Two chambers of $\mathscr {X}$ are *i*-*adjacent* if they are *i*-adjacent in some apartment. For $i\in I$ and for a chamber *C* of $\mathscr {X}$, let $q_i(C)$ be equal to

$$\begin{aligned} q_i(C) = {|{\{C^{\prime } \in \mathscr {X}: C^{\prime } \sim _i C\}} |} - 1. \end{aligned}$$

It may be proved that $q_i(C)$ is independent of *C* and of *i*. Denote the common value by *q*, and assume local finiteness: $q<\infty$. Any *edge* of $\mathscr {X}$, i.e., any 1-simplex, is contained in precisely $q+1$ chambers.

It follows from the axioms that the ball of radius one about any vertex *x* of $\mathscr {X}$ is made up of *x* itself, which is of one type, $q^2+q+1$ vertices of a second type, and a further $q^2+q+1$ vertices of the third type. Moreover, adjacency between vertices of the second and third types makes them into, respectively, the points and the lines of a finite projective plane.

A subcomplex $\mathscr {S}$ is called a *sector* of $\mathscr {X}$ if it is a sector in some apartment. Two sectors are called *equivalent* if they contain a common subsector. Let $\Omega$ denote the set of equivalence classes of sectors. If *x* is a vertex of $\mathscr {X}$ and $\omega \in \Omega$, there is a unique sector denoted $[x,\omega ]$ which has base vertex *x* and represents $\omega$.

Given any two points $\omega$ and $\omega ^{\prime } \in \Omega$, one can find two sectors representing them which lie in a common apartment. If that apartment is unique, we say that $\omega$ and $\omega ^{\prime }$ are *opposite*, and denote the unique apartment by $[\omega ,\omega ^{\prime }]$. In fact, $\omega$ and $\omega '$ are opposite precisely when the two sectors in the common apartment point in opposite directions in the Euclidean sense.

### Filtrations

We fix once and for all an origin vertex $O \in \mathscr {X}$ and a point $\omega _0 \in \Omega$. Choose *O* so that it has the same type as the origin of $\Sigma$. Let $\mathscr {S}_0=[O,\omega _0]$ be the sector representing $\omega _0$ with base vertex *O*. By $\Omega _0$, we denote the subset of $\Omega$ consisting of $\omega$’s opposite to $\omega _0$. For purposes of motivation only, we recall that if $\mathscr {X}$ is the building of ${\text {GL}}(3,\mathbb {Q}_p)$, then $\Omega _0$ can be identified with the *p*-adic Heisenberg group (see Appendix 1 for details).

Let $\mathscr {A}_0$ be any apartment containing $\mathscr {S}_0$. By $\psi$, we denote the type-preserving isomorphism between $\mathscr {A}_0$ and $\Sigma$ such that $\psi (\mathscr {S}_0) = -S_0$. We set $\rho = \psi \circ \rho _0$ where $\rho _0$ is the retraction from $\mathscr {X}$ to $\mathscr {A}_0$. With these definitions, $\rho :\mathscr {X}\rightarrow \Sigma$ is a type-preserving simplicial map, and for any $\omega \in \Omega _0$ the apartment $[\omega ,\omega _0]$ maps bijectively to $\Sigma$ with $\omega _0$ mapping to the bottom (of Fig. 1) and $\omega$ mapping to the top.

For any vertex *x* of $\mathscr {X}$, define the subset $E_x \subset \Omega _0$ to consist of all $\omega$’s such that *x* belongs to $[\omega , \omega _0]$; an equivalent condition is that $[x,\omega _0]\subseteq [\omega ,\omega _0]$. Fix $\lambda \in P$. By $\mathcal {F}_\lambda$, we denote the $\sigma$-field generated by sets $E_x$ for $x \in \mathscr {X}$ with $\rho (x) = \lambda$. There are countably many such *x*, and the corresponding sets $E_x$ are mutually disjoint, and hence, $\mathcal {F}_\lambda$ is a countably generated atomic $\sigma$-field.

Let $\preceq$ denote the partial order on *P* where $\lambda \preceq \mu$ if and only if ${\langle \lambda - \mu , \alpha _1\rangle } \le 0$ and ${\langle \lambda - \mu , \alpha _2\rangle } \le 0$. If we draw and orient $\Sigma$ as in Fig. 1, then $\lambda \preceq \mu$ exactly when $\mu$ lies in the sector pointing upward from $\lambda$.

#### Proposition 2.1

If $$\lambda \preceq \mu $$, then $$\mathcal {F}_\lambda \subset \mathcal {F}_\mu $$.

#### Proof

Choose any vertex *x* so that $\rho (x)=\mu$. Because $\lambda \preceq \mu$, there is a unique vertex *y* in the sector $[x,\omega _0]$ so that $\rho (y)=\lambda$. For any $\omega \in E_x$, the apartment $[\omega ,\omega _0]$ contains *x*, and hence, it contains $[x,\omega _0]$, which hence contains *y*. This establishes that $E_x\subseteq E_y$. In other words, each atom of $\mathcal {F}_\mu$ is a subset of some atom of $\mathcal {F}_\lambda$. Hence, each atom of $\mathcal {F}_\lambda$ is a disjoint union of atoms of $\mathcal {F}_\mu$. $\square$

In fact, Proposition 2.1 says that $\left( {\mathcal {F}_\lambda }: {\lambda \in P}\right) =\left( {\mathcal {F}_{i\lambda _1+j\lambda _2}}: {i,j\in \mathbb {Z}}\right)$ is a two parameter filtration. Let

$$\begin{aligned} \mathcal {F} = \sigma \Big (\bigcup _{\lambda \in P} \mathcal {F}_\lambda \Big ). \end{aligned}$$

Let $\pi$ denote the unique $\sigma$-additive measure on $(\Omega _0, \mathcal {F})$ such that for $E_x \in \mathcal {F}_\lambda$

$$\begin{aligned} \pi (E_x) = q^{-2{\langle \lambda , \alpha _0\rangle }}. \end{aligned}$$

All $\sigma$-fields in this paper should be extended so as to include $\pi$-null sets.

A function $f(\omega )$ on $\Omega _0$ is $\mathcal {F}_\lambda$-measurable if it depends only on that part of the apartment $[\omega ,\omega _0]$ which retracts under $\rho$ to the sector pointing downward from $\lambda$. For $i, j \in \mathbb {Z}$ set

$$\begin{aligned}&\mathcal {F}_{i, \infty } = \sigma \Big (\bigcup _{j^{\prime } \in \mathbb {Z}} \mathcal {F}_{i \lambda _1 + j' \lambda _2}\Big ),&\mathcal {F}_{\infty , j} = \sigma \Big (\bigcup _{i^{\prime } \in \mathbb {Z}} \mathcal {F}_{i^{\prime }\lambda _1 + j \lambda _2}\Big ). \end{aligned}$$

A function $f(\omega )$ on $\Omega _0$ is $\mathcal {F}_{i,\infty }$-measurable (respectively $\mathcal {F}_{\infty ,j}$-measurable) if it depends only on that part of the apartment which retracts to a certain “lower” half-plane with boundary parallel to $\lambda _2$ (respectively $\lambda _1$).

If $\mathcal {F}^{\prime }$ is $\sigma$-subfield of $\mathcal {F}$, we denote by $\mathbb {E}[f | \mathcal {F}^{\prime }]$ the Radon–Nikodym derivative with respect to $\mathcal {F}^{\prime }$. If $\mathcal {F}^{\prime \prime }$ is another $\sigma$-subfield of $\mathcal {F}$, we write

$$\begin{aligned} \mathbb {E}[f | \mathcal {F}^{\prime } | \mathcal {F}^{\prime \prime }] = \mathbb {E}\big [\mathbb {E}[f | \mathcal {F}^{\prime }]\big | \mathcal {F}^{\prime \prime }\big ]. \end{aligned}$$

The $\sigma$-field generated by $\mathcal {F}^{\prime } \cup \mathcal {F}^{\prime \prime }$ is denoted by $\mathcal {F}^{\prime } \vee \mathcal {F}^{\prime \prime }$. We write $f_\lambda = \mathbb {E}_\lambda f = \mathbb {E}[f | \mathcal {F}_\lambda ]$ for $\lambda \in P$. If $\lambda \preceq \mu$, then it follows from Proposition 2.1 that $\mathbb {E}_\mu \mathbb {E}_\lambda =\mathbb {E}_\lambda \mathbb {E}_\mu =\mathbb {E}_\lambda$.

We note that the Cairoli–Walsh condition ($F_4$) introduced in [5] is not satisfied, i.e.

$$\begin{aligned} \mathbb {E}_{\lambda +\lambda _1} \mathbb {E}_{\lambda +\lambda _2} \ne \mathbb {E}_\lambda . \end{aligned}$$

Instead of ($F_4$), we have

#### Lemma 2.2

For a locally integrable function *f* on $\Omega _0$

2.1 $$\begin{aligned}&\mathbb {E}[f_{\lambda +\lambda _1} | \mathcal {F}_{\lambda +\lambda _2} | \mathcal {F}_{\lambda +\lambda _1}] =q^{-1} f_{\lambda +\lambda _1} -q^{-1} \mathbb {E}[f_{\lambda +\lambda _1} | \mathcal {F}_{\lambda +\lambda _1-\lambda _2} \vee \mathcal {F}_\lambda ] +f_\lambda , \qquad \quad \end{aligned}$$

2.2 $$\begin{aligned}&\big (\mathbb {E}_{\lambda +\lambda _2} \mathbb {E}_{\lambda +\lambda _1}\big )^2 = q^{-1} \mathbb {E}_{\lambda +\lambda _2} \mathbb {E}_{\lambda +\lambda _1} + (1-q^{-1}) \mathbb {E}_\lambda , \end{aligned}$$

and likewise if we exchange $\lambda _1$ and $\lambda _2$.

#### Proof

For the proof of (2.1) it is enough to consider $f = {\mathbf {1}_{{E_{p_1}}}}$ where $p_1$ is a vertex in $\mathscr {X}$ such that $\rho (p_1) = \lambda + \lambda _1$. Let $\mathscr {S}$ be the sector $[p_1,\omega _0]$ and let *x* be the unique vertex of $\mathscr {S}$ with $\rho (x) = \lambda$. The ball in $\mathscr {X}$ of radius 1 around *x* has the structure of a finite projective plane.

**Fig. 2:**
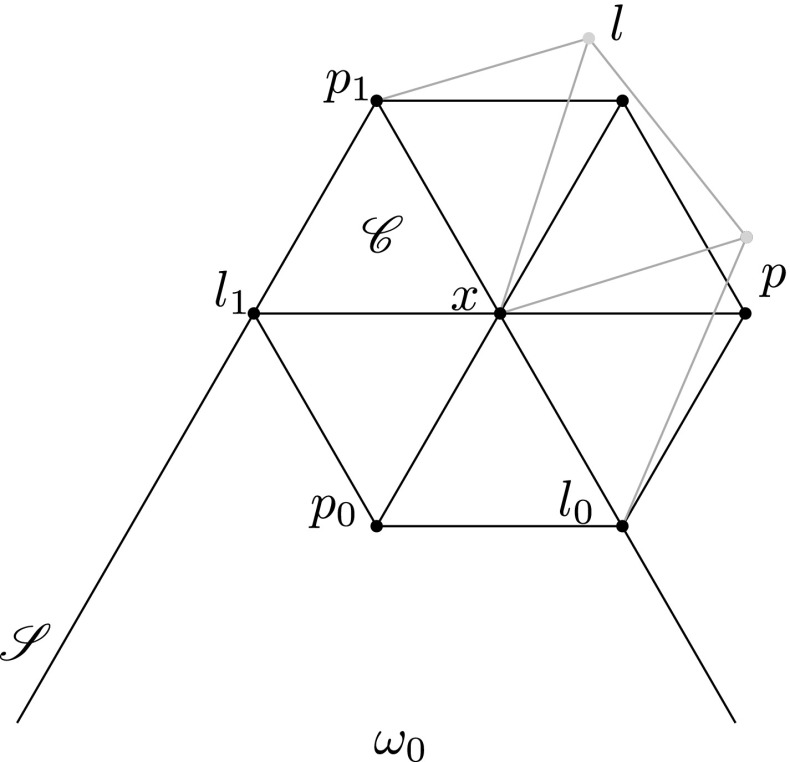
Residue of *x*

In Fig. 2, the spot marked *x* is for vertices of $\mathscr {X}$ which retract via $\rho$ to $\lambda$. Recall that $E_x$ is an atom of the $\sigma$-field $\mathcal {F}_\lambda$. The spot marked $p_1$ is for vertices retracting to $\lambda +\lambda _1$; the spot marked *l* is for vertices retracting to $\lambda +\lambda _2$; the spot marked $l_1$ is for vertices retracting to $\lambda +\lambda _1-\lambda _2$; etc. In the ball of radius 1 around *x*, only *x* itself retracts to the spot marked *x*. The line-type vertex known as $l_0$ is the only vertex in the ball retracting to its spot; *q* line-type vertices retract to the same spot as $l_1$; the remaining $q^2$ line-type vertices retract to the spot marked *l*. Likewise, $p_0$ is the unique point-type vertex of the ball retracting to its spot; *q* point-type vertices retract to the spot marked *p*; $q^2$ retract to the same spot as $p_1$. It follows that

$$\begin{aligned} \mathbb {E}[{\mathbf {1}_{{E_{p_1}}}} | \mathcal {F}_\lambda ] =q^{-2}{\mathbf {1}_{{E_x}}} =q^{-2}\sum _{p^{\prime }\not \sim l_0} {\mathbf {1}_{{E_{p^{\prime }}}}} =q^{-2}\sum _{l\not \sim p_0} {\mathbf {1}_{{E_l}}} \end{aligned}$$

and

$$\begin{aligned} \mathbb {E}[{\mathbf {1}_{{E_{p_1}}}} | \mathcal {F}_{\lambda +\lambda _1-\lambda _2}\vee \mathcal {F}_\lambda ] =q^{-1}{\mathbf {1}_{{E_x\cap E_{l_1}}}} =q^{-1}\sum _{\begin{array}{c} {p^{\prime }\sim l_1}\\ {p^{\prime }\not \sim l_0} \end{array}} {\mathbf {1}_{{E_{p^{\prime }}}}} \end{aligned}$$

where $p'$ runs through the point-type vertices of the ball, *l* runs through the line-type vertices of the ball, and $\sim$ stands for the incidence relation. We have

2.3 $$\begin{aligned} \mathbb {E}[{\mathbf {1}_{{E_{p_1}}}} | \mathcal {F}_{\lambda +\lambda _2}] = q^{-1} \sum _{\begin{array}{c} {l \sim p_1}\\ {l \not \sim p_0} \end{array}} {\mathbf {1}_{{E_l}}}. \end{aligned}$$

Therefore, we obtain

2.4 $$\begin{aligned} \begin{aligned} \mathbb {E}[{\mathbf {1}_{{E_{p_1}}}}|\mathcal {F}_{\lambda +\lambda _2}|\mathcal {F}_{\lambda +\lambda _1}]&= q^{-2} \sum _{\begin{array}{c} {l \sim p_1}\\ {l \not \sim p_0} \end{array}} \sum _{\begin{array}{c} {p^{\prime } \sim l}\\ {p^{\prime } \not \sim l_0} \end{array}} {\mathbf {1}_{{E_{p^{\prime }}}}} =q^{-1} {\mathbf {1}_{{E_{p_1}}}} + q^{-2}\sum _{\begin{array}{c} {p^{\prime } \not \sim l_0}\\ {p^{\prime } \not \sim l_1} \end{array}} {\mathbf {1}_{{E_{p^{\prime }}}}} \\&=q^{-1}{\mathbf {1}_{{E_{p_1}}}} + q^{-2} \sum _{p^{\prime } \not \sim l_0} {\mathbf {1}_{{E_{p^{\prime }}}}} -q^{-2} \sum _{\begin{array}{c} {p^{\prime } \sim l_1}\\ {p^{\prime }\not \sim l_0} \end{array}} {\mathbf {1}_{{E_{p^{\prime }}}}}, \end{aligned} \end{aligned}$$

which finishes the proof of (2.1). Applying one more average to the next to the last expression of (2.4), we get

$$\begin{aligned} \mathbb {E}[{\mathbf {1}_{{E_{p_1}}}} | \mathcal {F}_{\lambda +\lambda _2} | \mathcal {F}_{\lambda +\lambda _1} | \mathcal {F}_{\lambda +\lambda _2}] = q^{-2} \sum _{\begin{array}{c} {l \sim p_1}\\ {l \not \sim p_0} \end{array}} {\mathbf {1}_{{E_l}}} + q^{-3} \sum _{\begin{array}{c} {p^{\prime } \not \sim l_0}\\ {p^{\prime } \not \sim l_1} \end{array}} \sum _{\begin{array}{c} {l \sim p'}\\ {l \not \sim p_0} \end{array}} {\mathbf {1}_{{E_l}}}. \end{aligned}$$

For any line $l \not \sim p_0$, there are *q* points $p'$ such that $p' \sim l$ and $p^{\prime } \not \sim l_0$ and among them there is exactly one incident to $l_1$. Hence, in the last sum, each line $l \not \sim p_0$ appears $q-1$ times. Thus, we can write

$$\begin{aligned} q^{-3} \sum _{\begin{array}{c} {p^{\prime } \not \sim l_0}\\ {p^{\prime } \not \sim l_1} \end{array}} \sum _{\begin{array}{c} {l \sim p^{\prime }}\\ {l \not \sim p_0} \end{array}} = q^{-3} (q-1) \sum _{l \not \sim p_0} {\mathbf {1}_{{E_l}}} = (1-q^{-1}) \mathbb {E}[{\mathbf {1}_{{E_{p_1}}}} | \mathcal {F}_\lambda ] \end{aligned}$$

proving (2.2). $$\square $$

The following lemma describes the composition of projections on the same level.

#### Lemma 2.3

If $k, j \in \mathbb {Z}$ are such that $k \ge j \ge 0$ or $k \le j \le 0$ then

2.5 $$\begin{aligned} \mathbb {E}_{\lambda +k(\lambda _2-\lambda _1)} \mathbb {E}_\lambda = \mathbb {E}_{\lambda +k(\lambda _2-\lambda _1)} \mathbb {E}_{\lambda +j(\lambda _2-\lambda _1)} \mathbb {E}_\lambda . \end{aligned}$$

#### Proof

**Fig. 3:**
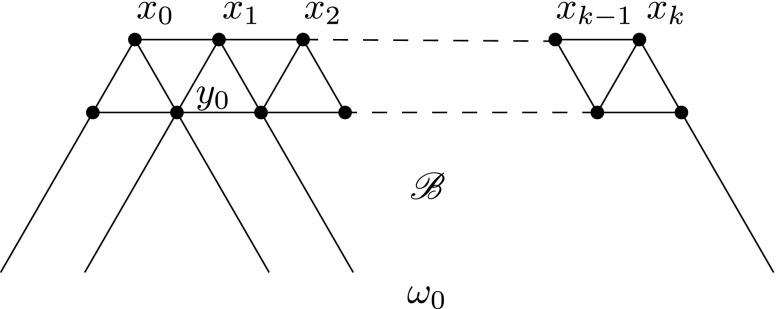
The complex $$\mathscr {B}$$

We carry out the proof for $k\ge j\ge 0$. For any $\omega \in \Omega _0$, there is a connected chain of vertices $\left( {x_i}: {0\le i\le k}\right) \subseteq [\omega ,\omega _0]$ with $\rho (x_i)=\lambda +k(\lambda _2-\lambda _1)$. Suppose, conversely, that $\left( {x_i}: {0\le i\le k}\right)$ is a connected chain of vertices and that $\rho (x_i)= \lambda +k(\lambda _2-\lambda _1)$. Construct a subcomplex $\mathscr {B}\subset \mathscr {X}$ by putting together $\left( {[x_i,\omega _0]}: {0\le i\le k}\right)$, the edges between the $x_i$’s and the triangles pointing downward from those edges to $\omega _0$. Referring to Fig. 3, the extra triangle pointing downward from the first edge has vertices $x_0$, $x_1$, and $y_0$. Note that $[x_0,\omega _0]\cap [x_1,\omega _0]=[y_0,\omega _0]$. Proceeding one step at a time, one may verify that the restriction of $\rho$ to $\mathscr {B}$ is an injection and that $\mathscr {B}$ and $\rho (\mathscr {B})$ are isomorphic complexes.

By basic properties of affine buildings, one knows it is possible to extend $\mathscr {B}$ to an apartment. Any such apartment will retract bijectively to $\Sigma$, and will be of the form form $[\omega ,\omega _0]$ where $\omega$ is the equivalence class represented by the upward pointing sectors of the apartment. Moreover, using the definition of $\pi$ one may calculate that

$$\begin{aligned} \pi (\{\omega \in \Omega _0 : \mathscr {B}\subseteq [\omega ,\omega _0]\}) =q^{-2{\langle \lambda , \alpha _0\rangle }-k} . \end{aligned}$$

The important point is that the measure of the set depends only on the level of $\lambda$ and the length of the chain.

Basic properties of affine buildings imply that any apartment containing $x_0$ and $x_k$ contains the entire chain. Hence,

$$\begin{aligned} \pi (E_{x_0}\cap E_{x_k}) =\pi (\{\omega \in \Omega _0 : \mathscr {B}\subseteq [\omega ,\omega _0]\}) =q^{-2{\langle \lambda , \alpha _0\rangle }-k} . \end{aligned}$$

Fix $x_0$. Proceeding one step at a time, one sees there are $q^k$ connected chains $\left( {x_i}: {0\le i\le k}\right)$ with $\rho (x_i)=\lambda +k(\lambda _2-\lambda _1)$. Consequently

$$\begin{aligned} \mathbb {E}_{\lambda +k(\lambda _2-\lambda _1)}{\mathbf {1}_{{x_0}}} =q^{-k}\sum _{\left( {x_i}: {0\le i\le k}\right) } {\mathbf {1}_{{x_k}}}. \end{aligned}$$

Likewise

$$\begin{aligned} \mathbb {E}_{\lambda +k(\lambda _2-\lambda _1)} \mathbb {E}_{\lambda +j(\lambda _2-\lambda _1)}{\mathbf {1}_{{x_0}}}&=q^{-j}\mathbb {E}_{\lambda +k(\lambda _2-\lambda _1)}\sum _{\left( {x_i}: {0\le i\le j}\right) } {\mathbf {1}_{{x_j}}} \\&=q^{-j}q^{-(k-j)}\sum _{\left( {x_i}: {0\le i\le j}\right) } \sum _{\left( {x_i}: {j\le i\le k}\right) } {\mathbf {1}_{{x_k}}}, \end{aligned}$$

which is the same thing. $\square$

Consider $\mathbb {E}_\lambda \mathbb {E}_\mu$. If $\lambda \preceq \mu$ then the product is equal to $\mathbb {E}_\lambda$; similarly if $\mu \preceq \lambda$. If $\lambda$ and $\mu$ are incomparable, the following lemma allows us to reduce to the case where $\lambda$ and $\mu$ are on the same level.

#### Lemma 2.4

Suppose $$\lambda \in P$$ and

$$\begin{aligned} \lambda ^{\prime }&= \lambda - i \lambda _1,&\mu&= \lambda ^{\prime } + k(\lambda _2 - \lambda _1),&\tilde{\mu }&= \mu +(\lambda _2-\lambda _1) \end{aligned}$$

for $i, k \in \mathbb {N}$. Then for any locally integrable function *f* on $\Omega _0$

2.6 $$\begin{aligned} \mathbb {E}[f | \mathcal {F}_\lambda | \mathcal {F}_\mu ]&= \mathbb {E}[f | \mathcal {F}_{\lambda ^{\prime }} | \mathcal {F}_\mu ], \end{aligned}$$

2.7 $$\begin{aligned} \mathbb {E}[f | \mathcal {F}_\mu | \mathcal {F}_\lambda ]&= \mathbb {E}[f | \mathcal {F}_\mu | \mathcal {F}_{\lambda ^{\prime }}], \end{aligned}$$

2.8 $$\begin{aligned} \mathbb {E}[f | \mathcal {F}_{\lambda } | \mathcal {F}_\mu \vee \mathcal {F}_{\tilde{\mu }}]&= \mathbb {E}[f | \mathcal {F}_{\lambda '} | \mathcal {F}_\mu ] \end{aligned}$$

2.9 $$\begin{aligned} \mathbb {E}[f | \mathcal {F}_\mu \vee \mathcal {F}_{\tilde{\mu }} | \mathcal {F}_\lambda ]&= \mathbb {E}[f | \mathcal {F}_\mu | \mathcal {F}_{\lambda '}] \end{aligned}$$

and likewise if we exchange $\lambda _1$ and $\lambda _2$.

#### Proof

We first prove (2.6) for $i=1$ and $k=1$. Because $\mathbb {E}[f|\mathcal {F}_{\lambda ^{\prime }}] =\mathbb {E}[f|\mathcal {F}_\lambda |\mathcal {F}_{\lambda ^{\prime }}]$, it is sufficient to consider $f={\mathbf {1}_{{E_{p_1}}}}$ where $\rho (p_1)=\lambda$. Use Fig. 2 to fix the notation, and note that if $p_1$ retracts to $\lambda$, then *x* retracts to $\lambda '$ and *p* to $\mu$. One calculates:

$$\begin{aligned} \mathbb {E}[{\mathbf {1}_{{E_{p_1}}}}| \mathcal {F}_\lambda | \mathcal {F}_\mu ] = \mathbb {E}[{\mathbf {1}_{{E_{p_1}}}}| \mathcal {F}_\mu ] = q^{-3} \sum _{\begin{array}{c} {p \sim l_0}\\ {p \ne p_0} \end{array}} {\mathbf {1}_{{E_p}}}&= q^{-2} \mathbb {E}[{\mathbf {1}_{{E_x}}} | \mathcal {F}_\mu ] \\&= \mathbb {E}[{\mathbf {1}_{{E_{p_1}}}} | \mathcal {F}_{\lambda ^{\prime }} | \mathcal {F}_\mu ]. \end{aligned}$$

**Fig. 4:**
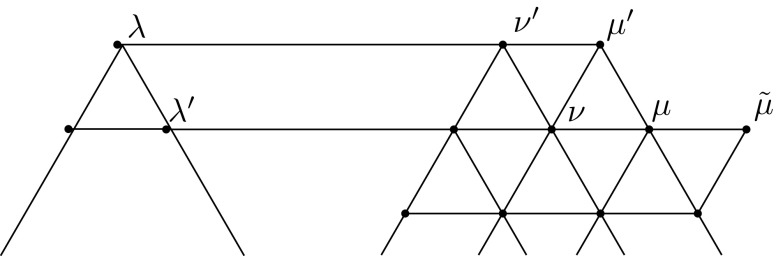
Notation used in Lemma 2.4

Next consider the case $i=1$, $k>1$. Set $\mu ^{\prime }=\mu +\lambda _1$, $\nu = \mu + \lambda _1 - \lambda _2$ and $\nu ' = \nu + \lambda _1$ (see Fig. 4). Since $\mathcal {F}_\mu$ is a subfield of $\mathcal {F}_{\mu ^{\prime }}$, we have

$$\begin{aligned} \mathbb {E}[f | \mathcal {F}_\lambda | \mathcal {F}_\mu ] = \mathbb {E}[f | \mathcal {F}_\lambda | \mathcal {F}_{\mu '} | \mathcal {F}_\mu ]. \end{aligned}$$

Thus, applying Lemma 2.3, we obtain

$$\begin{aligned} \mathbb {E}[f |\mathcal {F}_\lambda | \mathcal {F}_\mu ] =\mathbb {E}[f |\mathcal {F}_\lambda | \mathcal {F}_{\mu ^{\prime }} | \mathcal {F}_\mu ]&= \mathbb {E}[f | \mathcal {F}_\lambda | \mathcal {F}_{\nu '} | \mathcal {F}_{\mu '} | \mathcal {F}_\mu ] \\&= \mathbb {E}[f | \mathcal {F}_\lambda | \mathcal {F}_{\nu '} | \mathcal {F}_\mu ] = \mathbb {E}[f | \mathcal {F}_\lambda | \mathcal {F}_{\nu } | \mathcal {F}_\mu ] \end{aligned}$$

where in the last step we have used the case $k = 1$. Now apply induction on *k* and Lemma 2.3 again to get

$$\begin{aligned} \mathbb {E}[f | \mathcal {F}_\lambda | \mathcal {F}_{\nu } | \mathcal {F}_\mu ] = \mathbb {E}[f | \mathcal {F}_{\lambda ^{\prime }} | \mathcal {F}_{\nu } | \mathcal {F}_\mu ] = \mathbb {E}[f | \mathcal {F}_{\lambda ^{\prime }} | \mathcal {F}_\mu ] . \end{aligned}$$

To extend to the case $i>1$, use induction on *i* and observe that

$$\begin{aligned} \mathbb {E}[f | \mathcal {F}_\lambda | \mathcal {F}_\mu ] =\mathbb {E}[f | \mathcal {F}_\lambda | \mathcal {F}_{\mu ^{\prime }} | \mathcal {F}_\mu ]&=\mathbb {E}[f | \mathcal {F}_{\lambda ^{\prime } + \lambda _1} | \mathcal {F}_{\mu '}|\mathcal {F}_\mu ] \\&=\mathbb {E}[f | \mathcal {F}_{\lambda ^{\prime } + \lambda _1} | \mathcal {F}_\mu ] =\mathbb {E}[f | \mathcal {F}_{\lambda ^{\prime }} | \mathcal {F}_\mu ]. \end{aligned}$$

The proof of (2.8) is analogous, starting with the case $i=1$, $k=0$. Identity that (2.6) can be read as $\mathbb {E}_\mu \mathbb {E}_\lambda = \mathbb {E}_\mu \mathbb {E}_{\lambda '}$. The expectation operators are orthogonal projections with respect to the usual inner product, and taking adjoints gives $\mathbb {E}_\lambda \mathbb {E}_\mu = \mathbb {E}_{\lambda '} \mathbb {E}_\mu$ which is (2.7). To be more precise, one takes the inner product of either side of (2.7) with some nice test function, applies self-adjointness, and reduces to (2.6). Likewise, (2.9) follows from (2.8). $\square$

#### Lemma 2.5

Suppose $\lambda = i \lambda _1 + j \lambda _2$, $\mu = \lambda + k(\lambda _1 - \lambda _2)$. Then for any locally integrable function *f* on $\Omega _0$

$$\begin{aligned} \mathbb {E}[f | \mathcal {F}_\mu | \mathcal {F}_\lambda ] = {\left\{ \begin{array}{ll} \mathbb {E}[f | \mathcal {F}_\mu | \mathcal {F}_{i,\infty }] &{} \text {if } k \ge 0,\\ \mathbb {E}[f | \mathcal {F}_\mu | \mathcal {F}_{\infty ,j}] &{} \text {if } k \le 0 . \end{array}\right. } \end{aligned}$$

#### Proof

Suppose $k \ge 0$. By Lemma 2.4 for any $j' \ge 0$, we have

$$\begin{aligned} \mathbb {E}_\mu \mathbb {E}_{\lambda + j^{\prime } \lambda _2} = \mathbb {E}_\mu \mathbb {E}_\lambda . \end{aligned}$$

So if *g* is $\mathcal {F}_{\lambda + j^{\prime } \lambda _2}$-measurable and compactly supported, then

$$\begin{aligned} \begin{aligned} {\langle g, \mathbb {E}_{i,\infty }\mathbb {E}_\mu f\rangle } ={\langle \mathbb {E}_\mu \mathbb {E}_{i,\infty } g, f\rangle }&={\langle \mathbb {E}_\mu g, f\rangle } \\&={\langle \mathbb {E}_\mu \mathbb {E}_{\lambda +j^{\prime }\lambda _2} g, f\rangle } \\&={\langle \mathbb {E}_\mu \mathbb {E}_\lambda g, f\rangle } ={\langle g, \mathbb {E}_\lambda \mathbb {E}_\mu f\rangle }. \end{aligned} \end{aligned}$$

The test functions *g* which we use are sufficient to distinguish between one $\mathcal {F}_{i,\infty }$-measurable function and another. Since $\mathbb {E}_{i,\infty }\mathbb {E}_\mu f$ and $\mathbb {E}_\lambda \mathbb {E}_\mu f$ are both $\mathcal {F}_{i,\infty }$-measurable, the proof is done.

## Littlewood-Paley Theory

### Maximal Functions

The natural maximal function $M^*$ for a locally integrable function *f* on $\Omega _0$ is defined by

$$\begin{aligned} M^* f = \max _{\lambda \in P} {|{f_\lambda } |}. \end{aligned}$$

In addition, we define two auxiliary single-parameter maximal functions

$$\begin{aligned} L^* f&= \max _{i \in \mathbb {Z}} \mathbb {E}[\,|f|\, |\, \mathcal {F}_{i, \infty }],&R^* f&= \max _{j \in \mathbb {Z}} \mathbb {E}[\,|f|\, |\, \mathcal {F}_{\infty ,j}].&\end{aligned}$$

#### Lemma 3.1

Let $\lambda \in P$ and $k \in \mathbb {N}$. For any non-negative locally integrable function *f* on $\Omega _0$

$$\begin{aligned} \big (\mathbb {E}_{\lambda +k \lambda _2} \mathbb {E}_{\lambda +k\lambda _1}\big )^2 f \ge (1-q^{-1}) \mathbb {E}_\lambda f. \end{aligned}$$

#### Proof

**Fig. 5:**
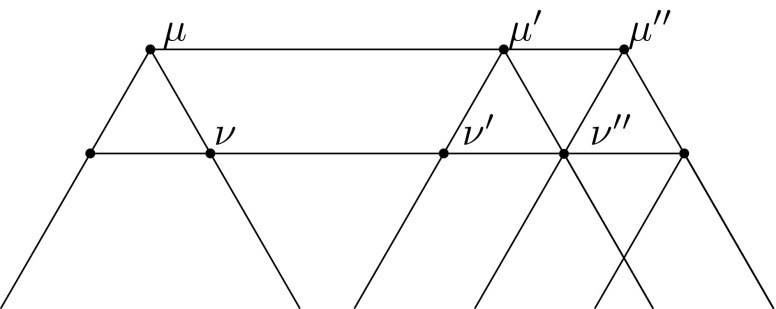
Notation used in Lemma 3.1

We may assume $\lambda = 0$. Let us define (see Fig. 5)

$$\begin{aligned} \mu&= k \lambda _1,&\mu '&= \lambda _1 + (k-1) \lambda _2,&\mu ''&= k \lambda _2,\\ \nu&= (k-1) \lambda _1,&\nu '&= \lambda _1 + (k-2) \lambda _2,&\nu ''&= (k-1)\lambda _2. \end{aligned}$$

We show

3.1 $$\begin{aligned} \mathbb {E}_{\mu ^{\prime \prime }} \mathbb {E}_{\mu } \mathbb {E}_{\mu ^{\prime \prime }} \mathbb {E}_{\mu } - q^{-1} \mathbb {E}_{\mu ^{\prime \prime }} \mathbb {E}_{\mu } \mathbb {E}_{\mu ^{\prime }} \mathbb {E}_{\mu } = \mathbb {E}_{\nu ^{\prime \prime }} \mathbb {E}_{\nu } \mathbb {E}_{\nu ^{\prime \prime }} \mathbb {E}_{\nu } - q^{-1} \mathbb {E}_{\nu ^{\prime \prime }} \mathbb {E}_{\nu } \mathbb {E}_{\nu ^{\prime }} \mathbb {E}_{\nu }. \end{aligned}$$

Let $g = \mathbb {E}[f| \mathcal {F}_{\mu }]$. By two applications of Lemma 2.3, we can write

$$\begin{aligned} \mathbb {E}[g | \mathcal {F}_{\mu ^{\prime \prime }} | \mathcal {F}_{\mu }] =\mathbb {E}[g | \mathcal {F}_{\mu ^{\prime }} | \mathcal {F}_{\mu ^{\prime \prime }} | \mathcal {F}_{\mu ^{\prime }} | \mathcal {F}_{\mu }] \end{aligned}$$

and by Lemma 2.2

$$\begin{aligned} \mathbb {E}[g | \mathcal {F}_{\mu ^{\prime }} | \mathcal {F}_{\mu ^{\prime \prime }} | \mathcal {F}_{\mu ^{\prime }}]= & {} q^{-1} \mathbb {E}[g | \mathcal {F}_{\mu ^{\prime }}] + \mathbb {E}[g | \mathcal {F}_{\nu ^{\prime \prime }}]\\&- q^{-1} \mathbb {E}[g |\mathcal {F}_{\mu ^{\prime }} | \mathcal {F}_{\nu ^{\prime }} \vee \mathcal {F}_{\nu ^{\prime \prime }}]. \end{aligned}$$

Hence,

$$\begin{aligned}&\mathbb {E}[g | \mathcal {F}_{\mu ^{\prime \prime }} | \mathcal {F}_{\mu } | \mathcal {F}_{\mu ^{\prime \prime }}] -q^{-1} \mathbb {E}[g | \mathcal {F}_{\mu '} | \mathcal {F}_{\mu } | \mathcal {F}_{\mu ^{\prime \prime }}]\\&\quad =\mathbb {E}[g | \mathcal {F}_{\nu ^{\prime \prime }} | \mathcal {F}_{\mu } | \mathcal {F}_{\mu ^{\prime \prime }}] -q^{-1} \mathbb {E}[g | \mathcal {F}_{\mu ^{\prime }} | \mathcal {F}_{\nu ^{\prime }} \vee \mathcal {F}_{\nu ^{\prime \prime }} |\mathcal {F}_{\mu } | \mathcal {F}_{\mu ^{\prime \prime }}]. \end{aligned}$$

By repeated application of Lemma 2.4, we have

$$\begin{aligned} \mathbb {E}[g | \mathcal {F}_{\nu ^{\prime \prime }} | \mathcal {F}_{\mu } | \mathcal {F}_{\mu ^{\prime \prime }}] =\mathbb {E}[f | \mathcal {F}_{\mu } | \mathcal {F}_{\nu ^{\prime \prime }} | \mathcal {F}_{\mu } | \mathcal {F}_{\mu ^{\prime \prime }}] =\mathbb {E}[f | \mathcal {F}_{\nu } | \mathcal {F}_{\nu ^{\prime \prime }} | \mathcal {F}_{\nu } | \mathcal {F}_{\nu ^{\prime \prime }}] \end{aligned}$$

and

$$\begin{aligned} \mathbb {E}[g | \mathcal {F}_{\mu ^{\prime }} | \mathcal {F}_{\nu ^{\prime }} \vee \mathcal {F}_{\nu ^{\prime \prime }} | \mathcal {F}_{\mu } | \mathcal {F}_{\mu ^{\prime \prime }}]&=\mathbb {E}[f | \mathcal {F}_\mu | \mathcal {F}_{\mu ^{\prime }} | \mathcal {F}_{\nu ^{\prime }} \vee \mathcal {F}_{\nu ^{\prime \prime }} | \mathcal {F}_{\mu } | \mathcal {F}_{\mu ^{\prime \prime }}] \\&= \mathbb {E}[f | \mathcal {F}_{\nu } | \mathcal {F}_{\nu ^{\prime }} | \mathcal {F}_{\nu } | \mathcal {F}_{\nu ^{\prime \prime }}] \end{aligned}$$

which finishes the proof of (3.1). By iteration of (3.1), we obtain

$$\begin{aligned}&\mathbb {E}_{\mu ^{\prime \prime }} \mathbb {E}_{\mu } \mathbb {E}_{\mu ^{\prime \prime }} \mathbb {E}_{\mu } - q^{-1} \mathbb {E}_{\mu ^{\prime \prime }} \mathbb {E}_{\mu } \mathbb {E}_{\mu ^{\prime }} \mathbb {E}_{\mu }\\&\qquad = \mathbb {E}_{\lambda _2} \mathbb {E}_{\lambda _1} \mathbb {E}_{\lambda _2} \mathbb {E}_{\lambda _1} - q^{-1} \mathbb {E}_{\lambda _2} \mathbb {E}_{\lambda _1} \mathbb {E}_{\lambda _1} \mathbb {E}_{\lambda _1} \end{aligned}$$

which together with Lemma 2.2 implies

$$\begin{aligned} \mathbb {E}_{\mu ^{\prime \prime }} \mathbb {E}_{\mu } \mathbb {E}_{\mu ^{\prime \prime }} \mathbb {E}_{\mu } = q^{-1} \mathbb {E}_{\mu ^{\prime \prime }} \mathbb {E}_{\mu } \mathbb {E}_{\mu '}\mathbb {E}_{\mu } + (1-q^{-1}) \mathbb {E}_0. \end{aligned}$$

$$\square $$

#### Theorem 1

For each $p \in (1, \infty ]$ there is $C_p > 0$ such that

3.2 $$\begin{aligned} {\left||L^* f \right||}_{L^p} \le C_p {\left||f \right||}_{L^p},\qquad {\left||R^* f \right||}_{L^p} \le C_p {\left||f \right||}_{L^p}, \end{aligned}$$

3.3 $$\begin{aligned} {\left||M^* f \right||}_{L^p} \le C_p {\left||f \right||}_{L^p}. \end{aligned}$$

#### Proof

Inequalities (3.2) are two instances of Doob’s well-known maximal inequality for single parameter martingales (see e.g. [15]). To show (3.3), consider a non-negative $f \in L^p(\Omega _0, \mathcal {F}_\mu )$. Fix $\lambda \in P$. Since $f \in L^p(\Omega _0, \mathcal {F}_{\mu '})$ for any $\mu ' \succeq \mu$ we may assume $\mu \succeq \lambda$. Let

$$\begin{aligned} \nu = \lambda + {\langle \mu - \lambda , \alpha _0\rangle } \lambda _1, \qquad \nu '' = \lambda + {\langle \mu - \lambda , \alpha _0\rangle } \lambda _2. \end{aligned}$$

By Lemma 3.1,

$$\begin{aligned} (1- q^{-1}) \mathbb {E}_\lambda f \le \mathbb {E}_{\nu ^{\prime \prime }} \mathbb {E}_{\nu } \mathbb {E}_{\nu ^{\prime \prime }} \mathbb {E}_{\nu } f. \end{aligned}$$

If $\lambda = i \lambda _1 + j \lambda _2$, then repeated application of Lemma 2.5 gives

$$\begin{aligned} \mathbb {E}_{\nu ^{\prime \prime }} \mathbb {E}_{\nu } \mathbb {E}_{\nu ^{\prime \prime }} \mathbb {E}_{\nu } f = \mathbb {E}_{\nu ^{\prime \prime }} \mathbb {E}_{\nu } \mathbb {E}_{\nu ^{\prime \prime }} \mathbb {E}_{\nu } \mathbb {E}_\mu f&= \mathbb {E}[f | \mathcal {F}_{\infty , j} | \mathcal {F}_{i, \infty } | \mathcal {F}_{\infty , j} | \mathcal {F}_{i, \infty } ] \\&\le L^* R^* L^* R^* f. \end{aligned}$$

By taking the supremum over $\lambda \in P$, we get

$$\begin{aligned} (1 - q^{-1}) M^* f \le L^* R^* L^* R^* f. \end{aligned}$$

Hence, by (3.2), we obtain (3.3) for $f \in L^p(\Omega _0, \mathcal {F}_\mu )$. Finally, a standard Fatou’s lemma argument establishes the theorem for arbitrary $f \in L^p(\Omega _0)$.

### Square Function

Let *f* be a locally integrable function on $\Omega _0$. Given $i, j \in \mathbb {Z}$, we define projections

$$\begin{aligned} L_i f = \mathbb {E}[f | \mathcal {F}_{i, \infty }] - \mathbb {E}[f | \mathcal {F}_{i-1, \infty }], \quad R_j f = \mathbb {E}[f | \mathcal {F}_{\infty , j}] - \mathbb {E}[f | \mathcal {F}_{\infty , j-1}]. \end{aligned}$$

Note that $L_i$ (respectively $R_j$) is the martingale difference operator for the filtration $\left( {\mathcal {F}_{i, \infty }}: {i \in \mathbb {Z}}\right)$ (respectively $\left( {\mathcal {F}_{\infty , j}}: {j \in \mathbb {Z}}\right)$). For $\lambda = i \lambda _1 + j \lambda _2$, we set

$$\begin{aligned} D_\lambda f = L_i R_j f, \qquad D_\lambda ^\star f = R_j L_i f. \end{aligned}$$

The following development is inspired by that of Stein and Street in [17]. We start by defining the corresponding square function.

$$\begin{aligned} \mathcal {S}f = \Big (\sum _{\lambda \in P} {|{D_\lambda f} |}^2 \Big )^{1/2}. \end{aligned}$$

We will also need its dual counterpart

$$\begin{aligned} \mathcal {S}^\star f = \Big (\sum _{\lambda \in P} {|{D_\lambda ^\star f} |}^2 \Big )^{1/2}. \end{aligned}$$

#### Theorem 2

For every $p \in (1, \infty )$ there is $C_p > 1$ such that

$$\begin{aligned} C_p^{-1} {\left||f \right||}_{L^p}&\le {\left||\mathcal {S}f \right||}_{L^p} \le C_p {\left||f \right||}_{L^p},&C_p^{-1} {\left||f \right||}_{L^p}&\le {\left||\mathcal {S}^\star f \right||}_{L^p} \le C_p {\left||f \right||}_{L^p}. \end{aligned}$$

Moreover, on $L^2(\Omega _0)$ square functions $\mathcal {S}$ and $\mathcal {S}^\star$ preserve the norm.

#### Proof

Since

$$\begin{aligned} S_L(f) = \Big (\sum _{i \in \mathbb {Z}} {|{L_i f} |}^2\Big )^{1/2} \quad \text {and}\quad S_R(f) = \Big (\sum _{j \in \mathbb {Z}} {|{R_j f} |}^2\Big )^{1/2} \end{aligned}$$

preserve the norm on $L^2(\Omega _0)$, we have

3.4 $$\begin{aligned} \begin{aligned} \int \sum _{i,j \in \mathbb {Z}} {|{L_i R_j f} |}^2 {\, \mathrm d}\pi&= \sum _{j \in \mathbb {Z}} \int \sum _{i \in \mathbb {Z}} {|{L_i R_j f} |}^2 {\, \mathrm d}\pi \\&= \sum _{j \in \mathbb {Z}} \int {|{R_j f} |}^2 d\pi = \int {|{f} |}^2 {\, \mathrm d}\pi . \end{aligned} \end{aligned}$$

Hence, $\mathcal {S}$ preserves the norm.

For $p \ne 2$, we use the two-parameter Khintchine inequality (see [12]) and bounds on single parameter martingale transforms (see [2, 15, 18]). Let $\left( {\epsilon _i}: {i \in \mathbb {Z}}\right)$ and $(\epsilon ^{\prime }_j : j \in \mathbb {Z})$ be sequences of real numbers, with absolute values bounded above by 1. For $N \in \mathbb {N}$, we consider the operator

$$\begin{aligned} T_N = \sum _{{|{i} |}, {|{j} |} \le N} \epsilon _i \epsilon _j' D_{i\lambda _1 + j \lambda _2} \end{aligned}$$

which may be written as a composition $\mathcal {L}_N \mathcal {R}_N$ where

$$\begin{aligned} \mathcal {L}_N = \sum _{{|{i} |} \le N} \epsilon _i L_i, \quad \mathcal {R}_N = \sum _{{|{j} |} \le N} \epsilon _j' R_j. \end{aligned}$$

Since by Burkholder’s inequality (see [2, 15]) the operators $\mathcal {R}_N$ and $\mathcal {L}_N$ are bounded on $L^p(\Omega _0)$ with bounds uniform in *N*, we have

$$\begin{aligned} {\left||T_N f \right||}_{L^p} \lesssim {\left||f \right||}_{L^p}. \end{aligned}$$

Setting $r_k$ to be the Rademacher function, by Khintchine’s inequality, we get

$$\begin{aligned}&\int \Big (\sum _{{|{i} |}, {|{j} |} \le N} {|{D_{i\lambda _1 + j \lambda _2} f} |}^2 \Big )^{p/2} {\, \mathrm d}\pi \\&\quad \lesssim \int \int _0^1 \int _0^1 \Big |\sum _{{|{i} |}, {|{j} |} \le N} r_i(s) r_j(t) D_{i \lambda _1 +j \lambda _2} f \Big |^p {\, \mathrm d}s{\, \mathrm d} t {\, \mathrm d}\pi , \end{aligned}$$

which is bounded by ${\left||f \right||}_{L^p}^p$. Finally, let *N* approach infinity and use the monotone convergence theorem to get

$$\begin{aligned} ||\mathcal {S}f ||_{L^p} \lesssim ||f ||_{L^p}. \end{aligned}$$

For the opposite inequality, we take $f \in L^p(\Omega _0) \cap L^2(\Omega _0)$ and $g \in L^{p^{\prime }}(\Omega _0) \cap L^2(\Omega _0)$ where $1/p'+1/p = 1$. By polarization of (3.4) and the Cauchy–Schwarz and Hölder inequalities, we obtain

$$\begin{aligned} {\langle f, g\rangle } = \int \sum _{\lambda \in P} D_\lambda f \overline{D_\lambda g} {\, \mathrm d}\pi \le \langle \mathcal {S}f, \mathcal {S}g \rangle \le {\left||\mathcal {S}f \right||}_{L^p} {\left||\mathcal {S}g \right||}_{L^{p^{\prime }}} \lesssim {\left||\mathcal {S}f \right||}_{L^p} {\left||g \right||}_{L^{p'}}. \end{aligned}$$

$$\square $$

Given a set $\{v_\lambda : \lambda \in P\}$ of vectors in a Banach space, we say that $\sum _{\lambda \in P} v_\lambda$ converges *unconditionally* if, whenever we choose a bijection $\phi : \mathbb {N}\rightarrow P$,

$$\begin{aligned} \sum _{n = 1}^\infty v_{\phi (n)} \qquad \text {exists, and is independent of }\phi . \end{aligned}$$

Equivalently, we may ask that for any increasing, exhaustive sequence $\left( {F_N}: {N \in \mathbb {N}}\right)$ of finite subsets of *P*, the limit

$$\begin{aligned} \lim _{N \rightarrow \infty } \sum _{\lambda \in F_N} v_\lambda \qquad \text {exists.} \end{aligned}$$

The following proposition provides a Calderón reproducing formula.

#### Proposition 3.2

For each $p \in (1, \infty )$ and any $f \in L^p(\Omega _0)$,

$$\begin{aligned} f = \sum _{\lambda \in P} D_\lambda D_\lambda ^\star f \end{aligned}$$

where the sum converges in $L^p(\Omega _0)$ unconditionally.

#### Proof

Fix an increasing and exhaustive sequence $\left( {F_N}: {N \in \mathbb {N}}\right)$ of finite subsets of *P*. Let

$$\begin{aligned} I_N(f) = \sum _{\lambda \in F_N} D_\lambda D_\lambda ^\star f. \end{aligned}$$

For $f \in L^p(\Omega _0)$ and $g \in L^{p^{\prime }}(\Omega _0)$, where $1/p + 1/{p^{\prime }} = 1$, we have

3.5 $$\begin{aligned} \begin{aligned} {|{{\langle I_N(f) - I_M(f), g\rangle }} |}&= \Big |\sum _{\lambda \in F_N \setminus F_M} {\langle D_\lambda ^\star f, D_\lambda ^\star g\rangle } \Big |\\&\le \Big ||\Big ( \sum _{\lambda \in F_N \setminus F_M} (D_\lambda ^\star f)^2 \Big )^{1/2} \Big ||_{L^p} {\left||\mathcal {S}^\star (g) \right||}_{L^{p^{\prime }}}. \end{aligned} \end{aligned}$$

In particular,

$$\begin{aligned} {|{{\langle I_N(f), g\rangle }} |} \le {\left||\mathcal {S}^\star (f) \right||}_{L^p} {\left||\mathcal {S}^\star (g) \right||}_{L^{p^{\prime }}}, \end{aligned}$$

whence ${\left||I_N(f) \right||}_{L^p} \lesssim {\left||f \right||}_{L^p}$ uniformly in *N*. Consequently, it is enough to prove convergence for $f \in L^p(\Omega _0) \cap L^2(\Omega _0)$. From (3.5) and the bounded convergence theorem, it follows that for any positive $\epsilon$, ${\left||I_N(f) - I_M(f) \right||}_{L^p} \le \epsilon$ whenever *M* and *N* are large enough. This shows that the limit exists. Finally, for $g \in L^{p^{\prime }}(\Omega _0)\cap L^2(\Omega _0)$, the polarized version of (3.4) gives

$$\begin{aligned} \lim _{N \rightarrow \infty } {\langle I_N(f), g\rangle } = \lim _{N \rightarrow \infty } \sum _{\lambda \in F_N} {\langle D_\lambda ^\star f, D_\lambda ^\star g\rangle } = {\langle f, g\rangle }. \end{aligned}$$

$$\square $$

#### Theorem 3

Let $\left( {T_\lambda }: {\lambda \in P}\right)$ be a family of operators such that for some $\delta > 0$ and $p_0 \in (1, 2)$

3.6 $$\begin{aligned}&{{\left||{T_\lambda } \right||}_{L^{1} \rightarrow L^{1}}} \lesssim 1, \end{aligned}$$

3.7 $$\begin{aligned}&{{\left||{T_\mu T_\lambda ^\star } \right||}_{L^{2} \rightarrow L^{2}}} \lesssim q^{-\delta {|{\mu -\lambda } |}} \quad \text {and} \quad {{\left||{T_\mu ^\star T_\lambda } \right||}_{L^{2} \rightarrow L^{2}}} \lesssim q^{-\delta {|{\mu -\lambda } |}}, \end{aligned}$$

3.8 $$\begin{aligned}&{{\left||{D_\lambda T_\mu D_{\lambda ^{\prime }}} \right||}_{L^{2} \rightarrow L^{2}}} \lesssim q^{-\delta {|{\lambda -\mu } |}} q^{-\delta {|{\lambda '-\mu } |}}, \end{aligned}$$

3.9 $$\begin{aligned}&\big ||{\sup _{\lambda \in P} {|{T_\lambda f_\lambda } |}}\big ||_{L^{p_0}} \lesssim \big ||\sup _{\lambda \in P} {|{f_\lambda } |} \big ||_{L^{p_0}}. \end{aligned}$$

Then for any $p \in (p_0, 2]$ the sum $\sum _{\lambda \in P} T_\lambda$ converges unconditionally in the strong operator topology for operators on $L^p(\Omega _0)$.

#### Proof

First, recall that the Cotlar–Stein Lemma (see e.g. [16]) states that (3.7) implies the unconditional convergence of $\sum _{\lambda \in P} T_\lambda$ in the strong operator topology on $L^2(\Omega _0)$. Let $\left( {F_N}: {N \in \mathbb {N}}\right)$ be an arbitrary increasing and exhaustive sequence of finite subsets of *P*. For $N > 0$, we set

$$\begin{aligned} V_N&= \sum _{\mu \in F_N} T_\mu ,&I_N&= \sum _{\lambda \in F_N} D_\lambda D_\lambda ^\star . \end{aligned}$$

By (3.6), (3.7) and interpolation, each $T_\mu$ is bounded on $L^p$ for $p\in [1,2]$ and the same holds for the finite sum $V_N$. We consider $f \in L^p(\Omega _0)$ for $p \in (p_0, 2)$. By Proposition 3.2 and Theorem 2, we

$$\begin{aligned}&\big ||V_M I_N (f) \big ||_{L^p}\\&\quad \lesssim \big ||\mathcal {S}\big (V_M I_N(f) \big ) \big ||_{L^p} =\Big ||\Big ( \sum _{\mu \in F_M} \sum _{\lambda ^{\prime } \in F_N} D_\lambda T_\mu D_{\lambda ^{\prime }} D_{\lambda ^{\prime }}^\star f : \lambda \in P \Big ) \Big ||_{L^p(\ell ^2)}\\&\quad =\Big ||\Big ( \sum _{\gamma , \gamma ^{\prime } \in P} {\mathbf {1}_{{F_N}}}(\lambda + \gamma + \gamma ^{\prime }) {\mathbf {1}_{{F_M}}}(\lambda + \gamma ) D_\lambda T_{\lambda +\gamma } D_{\lambda +\gamma +\gamma ^{\prime }} D_{\lambda +\gamma +\gamma ^{\prime }}^\star f : \lambda \in P \Big ) \Big ||_{L^p(\ell ^2)}\\&\quad \le \sum _{\gamma , \gamma ^{\prime } \in P} \big ||\left( { {\mathbf {1}_{{F_N}}}(\lambda +\gamma +\gamma ^{\prime }) {\mathbf {1}_{{F_M}}}(\lambda +\gamma ) D_\lambda T_{\lambda +\gamma } D_{\lambda +\gamma +\gamma '} D_{\lambda +\gamma +\gamma '}^\star f}: {\lambda \in P}\right) \big ||_{L^p(\ell ^2)}. \end{aligned}$$

Finally, by change of variables, we get

$$\begin{aligned} \big ||V_M I_N(f) \big ||_{L^p} \lesssim \sum _{\gamma ,\gamma ^{\prime } \in P} \big ||\left( { D_{\lambda +\gamma +\gamma ^{\prime }} T_{\lambda +\gamma } D_\lambda D_\lambda ^\star f}: {\lambda \in F_N}\right) \big ||_{L^p(\ell ^2)}. \end{aligned}$$

Assuming there is $\delta _p > 0$ such that

3.10 $$\begin{aligned} {\left||\left( {D_{\lambda +\gamma +\gamma ^{\prime }} T_{\lambda +\gamma } D_\lambda f_\lambda }: {\lambda \in P}\right) \right||}_{L^p(\ell ^2)} \lesssim q^{-\delta _p ({|{\gamma } |} + {|{\gamma ^{\prime }} |})} {\left||\left( {f_\lambda }: {\lambda \in P}\right) \right||}_{L^p(\ell ^2)}\nonumber \\ \end{aligned}$$

we can estimate

3.11 $$\begin{aligned} \begin{aligned} \big ||V_M I_N(f) \big ||_{L^p}&\lesssim \sum _{\gamma , \gamma ^{\prime } \in P} q^{-\delta _p({|{\gamma } |} + {|{\gamma ^{\prime }} |})} {\left||\left( {D_\lambda ^\star f}: {\lambda \in F_N}\right) \right||}_{L^p(\ell ^2)} \\&\lesssim \Big ||\Big (\sum _{\lambda \in F_N} (D_\lambda ^\star f)^2\Big )^{1/2} \Big ||_{L^p}. \end{aligned} \end{aligned}$$

Theorem 2, Proposition 3.2 and (3.11) imply that the $V_M$ are uniformly bounded on $L^p$.

For the proof of (3.10), we consider an operator $\mathcal {T}$ defined for $\mathbf {f} \in L^p\big (\pi , \ell ^2(P)\big )$ by

$$\begin{aligned} \mathcal {T} {\vec {f}} = \left( {D_{\lambda +\gamma +\gamma ^{\prime }} T_{\lambda +\gamma } D_\lambda f_\lambda }: {\lambda \in P}\right) . \end{aligned}$$

Since ${{\left||{D_\lambda } \right||}_{L^{1} \rightarrow L^{1}}} \lesssim 1$ and ${{\left||{T_\mu } \right||}_{L^{1} \rightarrow L^{1}}} \lesssim 1$, we have

$$\begin{aligned} \big ||\mathcal {T} {\vec f\;} \big ||_{L^1(\ell ^1)} \lesssim \big ||\vec f\; \big ||_{L^1(\ell ^1)}. \end{aligned}$$

Also, by (3.8), we can estimate

$$\begin{aligned} \big \Vert \mathcal {T} \vec f\; \big ||_{L^2(\ell ^2)}^2 = \sum _{\lambda \in P} {\left||D_{\lambda +\gamma +\gamma ^{\prime }} T_{\lambda +\gamma } D_\lambda f_\lambda \right||}_{L^2}^2 \lesssim q^{-\delta ({|{\gamma } |}+{|{\gamma ^{\prime }} |})} \sum _{\lambda \in P} {\left||f_\lambda \right||}_{L^2}^2. \end{aligned}$$

Therefore, using interpolation between $L^{1}\left( \pi , \ell ^{1}(P) \right)$ and $L^{2}\left( \pi , \ell ^{2}(P) \right)$ we obtain that there is $\delta ^{\prime } > 0$ such that

$$\begin{aligned} \big ||\mathcal {T} {\vec f}\; \big ||_{L^{p_0}(\ell ^{p_0})} \lesssim q^{-\delta ^{\prime } ({|{\gamma } |}+{|{\gamma ^{\prime }} |})} \big ||\vec f\; \big ||_{L^{p_0}(\ell ^{p_0})}. \end{aligned}$$

Because ${|{D_\lambda g} |} \lesssim L^* R^*( {|{g} |} )$, and because Theorem 1 says that $L^*$ and $R^*$ are bounded on $L^{p_0}$, we know that $\left( {D_\lambda }: {\lambda \in P}\right)$ is bounded on $L^{p_0}(\pi , \ell ^\infty (P))$. Of course the same holds for $\left( {D_{\lambda +\gamma +\gamma '}}: {\lambda \in P}\right)$. Hence, by (3.9), we get

$$\begin{aligned} \big ||\mathcal {T} {\vec f}\; \big ||_{L^{p_0}(\ell ^\infty )} \lesssim \big ||\vec f\;\big ||_{L^{p_0}(\ell ^\infty )}. \end{aligned}$$

Next, interpolating between $L^{p_0}\left( \pi , \ell ^{p_0}(P) \right)$ and $L^{p_0}\left( \pi , \ell ^{\infty }(P) \right)$ gives a $\delta ^{\prime \prime } > 0$ such that

$$\begin{aligned} \big ||\mathcal {T} {\vec f}\; \big ||_{L^{p_0}(\ell ^2)} \lesssim q^{-\delta ^{\prime \prime }({|{\gamma } |} + {|{\gamma ^{\prime }} |})} \big ||\vec f\;\big ||_{L^{p_0}(\ell ^2)}. \end{aligned}$$

Finally, interpolating between $L^{p_0}\left( \pi , \ell ^{2}(P) \right)$ and $L^{2}\left( \pi , \ell ^{2}(P) \right)$, we obtain (3.10).

To complete the proof, we are going to show that $\left( {V_N f}: {N \in \mathbb {N}}\right)$ is a Cauchy sequence in $L^p(\Omega _0)$. Let us consider $g \in L^p(\Omega _0) \cap L^2(\Omega _0)$. Setting

$$\begin{aligned} a = \frac{2(p-p_0)}{4-p-p_0},\quad \text {and} \quad \tilde{p} = \frac{p + p_0}{2} \end{aligned}$$

and using the log-convexity of the $L^q$-norms, we get

$$\begin{aligned} \big ||V_M g - V_N g \big ||_{L^p}^p \le \big ||V_M g - V_N g \big ||_{L^2}^a \big ||V_M g - V_N g \big ||_{L^{\tilde{p}}}^{p-a}. \end{aligned}$$

Since $\left( {V_N g}: {N \in \mathbb {N}}\right)$ converges in $L^2(\Omega _0)$ and is uniformly bounded on $L^{\tilde{p}}(\Omega _0)$ it is a Cauchy sequence in $L^p(\Omega _0)$. For an arbitrary $f \in L^p(\Omega _0)$ use the density of *g*’s as above. We have

$$\begin{aligned} {\left||V_M f - V_N f \right||}_{L^p} \lesssim {\left||f - g \right||}_{L^p} + {\left||V_N g - V_M g \right||}_{L^p}. \end{aligned}$$

Thus, $\left( {V_N f}: {N\in \mathbb {N}}\right)$ also converges, and this finishes the proof of the theorem. $\square$

## Double Differences

The martingale transforms are expressed in terms of double differences defined for a martingale $f = \left( {f_\lambda }: {\lambda \in P}\right)$ as

$$\begin{aligned} d_\lambda f= f_{\lambda } - f_{\lambda -\lambda _1} - f_{\lambda - \lambda _2} + f_{\lambda -\lambda _1 - \lambda _2}. \end{aligned}$$

### Martingale Transforms

The following proposition is our key tool.

#### Proposition 4.1

Let $f \in L^2(\Omega _0)$ and $\lambda \in P$. If $f_{\lambda - j \lambda _1} = 0$ for $j \in \mathbb {N}$ then for each $k \ge j$

$$\begin{aligned} {\left||\mathbb {E}[f_\lambda | \mathcal {F}_{\lambda -k(\lambda _1-\lambda _2)}] \right||}_{L^2} \le 2 q^{-(k-j+1)/2}{\left||f_\lambda \right||}_{L^2}. \end{aligned}$$

Analogously, for $\lambda _1$ and $\lambda _2$ exchanged.

#### Proof

Suppose $j = 1$. We are going to show that if $f_{\lambda - \lambda _1} = 0$ then for all $k \ge 1$

4.1 $$\begin{aligned} {\left||\mathbb {E}[f_\lambda | \mathcal {F}_{\lambda - k(\lambda _1 - \lambda _2)} ] \right||}_{L^2} \le q^{-k/2} {\left||f_\lambda \right||}_{L^2}. \end{aligned}$$

Indeed, if $k = 1$ then by (2.1) of Lemma 2.2

$$\begin{aligned} \big ||\mathbb {E}[f_\lambda |\mathcal {F}_{\lambda -\lambda _1+\lambda _2}] \big ||_{L^2}^2&={\langle \mathbb {E}[f_\lambda | \mathcal {F}_{\lambda -\lambda _1+\lambda _2} | \mathcal {F}_\lambda ], f_\lambda \rangle } \\&= q^{-1} {\left||f_\lambda \right||}^2_{L^2} - q^{-1} \big ||\mathbb {E}[f_\lambda | \mathcal {F}_{\lambda -\lambda _1} \vee \mathcal {F}_{\lambda -\lambda _2}] \big ||_{L^2}^2. \end{aligned}$$

If $k > 1$, we use Lemma 2.3 to write

$$\begin{aligned} \mathbb {E}[f_\lambda | \mathcal {F}_{\lambda -k(\lambda _1-\lambda _2)}] = \mathbb {E}[f_\lambda |\mathcal {F}_{\lambda -(\lambda _1-\lambda _2)} | \mathcal {F}_{\lambda -k(\lambda _1-\lambda _2)}]. \end{aligned}$$

Since, by Lemma 2.4,

$$\begin{aligned} \mathbb {E}[f_\lambda | \mathcal {F}_{\lambda -(\lambda _1-\lambda _2)} | \mathcal {F}_{\lambda -\lambda _1 - (\lambda _1 - \lambda _2)}] = \mathbb {E}[f_\lambda | \mathcal {F}_{\lambda -\lambda _1} | \mathcal {F}_{\lambda -\lambda _1- (\lambda _1 - \lambda _2)}] = 0 \end{aligned}$$

we can use induction to obtain

$$\begin{aligned} {\left||\mathbb {E}[f_\lambda | \mathcal {F}_{\lambda -(\lambda _1-\lambda _2)} | \mathcal {F}_{\lambda -k(\lambda _1-\lambda _2)}] \right||}_{L^2}&\le q^{-(k-1)/2} \big ||\mathbb {E}[f_\lambda | \mathcal {F}_{\lambda -(\lambda _1-\lambda _2)}] \big ||_{L^2} \\&\le q^{-k/2} {\left||f_\lambda \right||}_{L^2}. \end{aligned}$$

Let us consider $j > 1$. For each $i = 0, 1, \ldots , j-1$, we set

$$\begin{aligned} g_i = f_{\lambda - i \lambda _1} - f_{\lambda - (i+1) \lambda _1}. \end{aligned}$$

By Lemma 2.4 and (4.1), we have

$$\begin{aligned} {\left||\mathbb {E}[g_i | \mathcal {F}_{\lambda -k(\lambda _1 - \lambda _2)}] \right||}_{L^2} ={\left||\mathbb {E}[g_i | \mathcal {F}_{\lambda - k(\lambda _1- \lambda _2) - i \lambda _2}] \right||}_{L^2}&\le q^{-(k-i)/2} {\left||g_i \right||}_{L^2} \\&\le q^{-(k-i)/2} {\left||f_\lambda \right||}_{L^2}. \end{aligned}$$

Hence,

$$\begin{aligned} \big ||\mathbb {E}[f_\lambda | \mathcal {F}_{\lambda - k(\lambda _1 - \lambda _2)}] \big ||_{L^2}&\le \sum _{i=0}^{j-1} \big ||\mathbb {E}[g_i | \mathcal {F}_{n- k(\lambda _1- \lambda _2)}] \big ||_{L^2} \\&\le \sum _{i = 0}^{j-1} q^{-(k-i)/2} {\left||f_\lambda \right||}_{L^2} \end{aligned}$$

which finishes the proof since

$$\begin{aligned} \sum _{i = 0}^{j-1} q^{i/2} \le 2 q^{(j-1)/2}. \end{aligned}$$

$$\square $$

We have the following

#### Proposition 4.2

For any $\lambda , \lambda ^{\prime }, \mu \in P$ and $m \ge 1$

$$\begin{aligned} {{\left||{D_\lambda d_\mu ^m D_{\lambda ^{\prime }}} \right||}_{L^{2} \rightarrow L^{2}}}&\lesssim q^{-{|{\mu -\lambda } |}/4} q^{-{|{\mu -\lambda ^{\prime }} |}/4},\\ {{\left||{d_\lambda ^m d_\mu ^m} \right||}_{L^{2} \rightarrow L^{2}}}&\lesssim q^{-{|{\lambda -\mu } |}/2}. \end{aligned}$$

#### Proof

We observe that for $f \in L^2(\Omega _0)$, $d_\mu f \in L^2(\pi , \mathcal {F}_\mu )$ and

4.2 $$\begin{aligned} \mathbb {E}[d_\mu f| \mathcal {F}_{\nu }]=0 \end{aligned}$$

whenever ${\langle \nu , \alpha _0\rangle } \le {\langle \mu , \alpha _0\rangle } - 2$. For the proof it is enough to analyze the case $\nu = \mu - 2 \lambda _2$. By Lemma 2.4, we can write

$$\begin{aligned} \mathbb {E}[f_{\mu -\lambda _1} | \mathcal {F}_{\mu - 2 \lambda _2}] = \mathbb {E}[f_{\mu -\lambda _1} | \mathcal {F}_{\mu -\lambda _1-\lambda _2} |\mathcal {F}_{\mu -2\lambda _2}] = \mathbb {E}[f_{\mu -\lambda _1-\lambda _2} | \mathcal {F}_{\mu -2\lambda _2}]. \end{aligned}$$

Suppose $\lambda = i \lambda _1 + j \lambda _2$. Let us consider $R_j d_\mu$. If $j \ge {\langle \mu , \alpha _2\rangle } + 1$ then $R_j d_\mu f = 0$. For $j \le {\langle \mu , \alpha _2\rangle } - 2$, in view of (4.2) we can use Proposition 4.1 to estimate

4.3 $$\begin{aligned} {\left||R_j d_\mu f \right||}_{L^2} \lesssim q^{-{\langle \mu - \lambda , \alpha _2\rangle }/2} {\left||d_\mu f \right||}_{L^2}. \end{aligned}$$

Next, if ${\langle \lambda , \alpha _0\rangle } \ge {\langle \mu , \alpha _0\rangle } + 2$ then $D_\lambda d_\mu f = 0$, because $d_\mu f$ is $\mathcal {F}_\mu$-measurable. For ${\langle \lambda , \alpha _0\rangle } \le {\langle \mu , \alpha _0\rangle } - 4$ and ${\langle \lambda , \alpha _2\rangle } \le {\langle \mu , \alpha _2\rangle }$, by Lemma 2.5, we can write $D_\lambda d_\mu f = L_i g$ where

$$\begin{aligned} g = \mathbb {E}[R_j d_\mu f | \mathcal {F}_{\nu }] \end{aligned}$$

and $\nu = ({\langle \mu , \alpha _0\rangle } - j)\lambda _1 + j \lambda _2$. By Lemma 2.5, we have

$$\begin{aligned} R_j d_\mu f = \mathbb {E}[d_\mu f | \mathcal {F}_\nu ] - \mathbb {E}[d_\mu f | \mathcal {F}_{\nu +\lambda _1-\lambda _2}]. \end{aligned}$$

We notice that by Lemma 2.4 and (4.2)

$$\begin{aligned} \mathbb {E}[d_\mu f | \mathcal {F}_{\nu } | \mathcal {F}_{\nu - 2 \lambda _1}] = \mathbb {E}[d_\mu f | \mathcal {F}_{\mu - 2\lambda _2} | \mathcal {F}_{\nu - 2 \lambda _1}] = 0. \end{aligned}$$

Similarly, one can show

$$\begin{aligned} \mathbb {E}[d_\mu f | \mathcal {F}_{\nu + \lambda _1 - \lambda _2} | \mathcal {F}_{\nu - 2\lambda _1}] = 0. \end{aligned}$$

Therefore, $\mathbb {E}[g | \mathcal {F}_{\nu - 2\lambda _1}] = 0$. Now, by Proposition 4.1, we obtain

4.4 $$\begin{aligned} {\left||L_i g \right||}_{L^2} \lesssim q^{-{\langle \nu -\lambda , \alpha _0\rangle }/2} {\left||R_j d_\mu f \right||}_{L^2}. \end{aligned}$$

Combining (4.4) with (4.3), we get

4.5 $$\begin{aligned} {\left||D_\lambda d_\mu f \right||}_{L^2} \lesssim q^{-{\langle \mu - \lambda , \alpha _0\rangle }/2} q^{-{\langle \mu - \lambda , \alpha _2\rangle }/2} {\left||d_\mu f \right||}_{L^2} \end{aligned}$$

since ${\langle \nu , \alpha _0\rangle } = {\langle \mu , \alpha _0\rangle }$. By analogous reasoning one can show the corresponding norm estimates for $D_{\lambda '}^\star d_\mu$. Hence, taking adjoint

4.6 $$\begin{aligned} {\left||d_\mu D_{\lambda ^{\prime }} f \right||}_{L^2} \lesssim q^{-{\langle \mu - \lambda ^{\prime }, \alpha _0\rangle }/2} q^{-{\langle \mu - \lambda ^{\prime }, \alpha _2\rangle }/2} {\left||f \right||}_{L^2}. \end{aligned}$$

Finally, (4.5) and (4.6) allow us to conclude the proof of the first inequality.

For the second, we may assume $0 \le {\langle \mu - \lambda , \alpha _0\rangle } \le 1$. Suppose ${\langle \mu - \lambda , \alpha _0\rangle } = 0$ and ${\langle \mu - \lambda , \alpha _2\rangle } \ge 2$. Since $d_\mu f \in L^2(\pi , \mathcal {F}_\mu )$, by (4.2) and Proposition 4.1

$$\begin{aligned} {\left||\mathbb {E}[d_\mu f| \mathcal {F}_\lambda ] \right||}_{L^2} \lesssim q^{-{\langle \mu - \lambda , \alpha _2\rangle }/2} {\left||d_\mu f \right||}_{L^2}. \end{aligned}$$

Similarly, we deal with the case ${\langle \mu - \lambda , \alpha _0\rangle } = 1$. We can assume ${\langle \mu - \lambda , \alpha _2\rangle } \ge 1$. By Lemma 2.4, we have

$$\begin{aligned} \mathbb {E}[d_\mu f | \mathcal {F}_\lambda ] = \mathbb {E}[d_\mu f | \mathcal {F}_{\mu -\lambda _2} | \mathcal {F}_\lambda ] = \mathbb {E}[f_{\lambda -\lambda _1-\lambda _2} - f_{\lambda -\lambda _1} | \mathcal {F}_\lambda ]. \end{aligned}$$

Hence, by Proposition 4.1,

$$\begin{aligned} {\left||\mathbb {E}[d_\mu f| \mathcal {F}_\lambda ] \right||}_{L^2} \lesssim q^{-{\langle \mu - \lambda , \alpha _2\rangle }/2} {\left||f \right||}_{L^2}. \end{aligned}$$

$$\square $$

Let $\left( {a_\lambda }: {\lambda \in P}\right)$ be an uniformly bounded *predictable* family of functions, i.e. each function $a_\lambda$ is measurable with respect to $\mathcal {F}_{\lambda -\lambda _1-\lambda _2}$ and

$$\begin{aligned} \sup _{\omega \in \Omega _0} {|{a_\lambda (\omega )} |} \le M. \end{aligned}$$

Predictability is the condition needed to ensure that $d_\lambda \big (a_\lambda f\big ) = a_\lambda d_\lambda f$. By Theorems 1 and 3, Proposition 4.2 and duality when $p > 2$, we get

#### Theorem 4

For each $p \in (1, \infty )$ and $m \in \mathbb {N}$ the series

$$\begin{aligned} \sum _{\lambda \in P} a_\lambda d_\lambda ^m \end{aligned}$$

converges unconditionally in the strong operator topology for the operators on $L^p(\Omega _0)$, and defines the operator with norm bounded by a constant multiply of

$$\begin{aligned} \sup _{\lambda \in P} \sup _{\omega \in \Omega _0} {|{a_\lambda (\omega )} |}. \end{aligned}$$

### Martingale Square Function

For a martingale $f = \left( {f_\lambda }: {\lambda \in P}\right)$ there is the natural square function defined by

$$\begin{aligned} S f = \Big (\sum _{\lambda \in P} (d_\lambda f)^2 \Big )^{1/2}. \end{aligned}$$

Although *S* does not preserve $L^2$ norm, we have

#### Theorem 5

For every $p \in (1, \infty )$ there is $C_p > 0$ such that

$$\begin{aligned} C_p^{-1} {\left||f \right||}_{L^p} \le {\left||S f \right||}_{L^p} \le C_p {\left||f \right||}_{L^p}. \end{aligned}$$

#### Proof

We start from proving the identity

4.7 $$\begin{aligned} d_\lambda ^4 - d_\lambda ^3 - q^{-1} d_\lambda ^2 + q^{-1} d_\lambda =0. \end{aligned}$$

Let us notice that

$$\begin{aligned} d_\lambda \mathbb {E}_{\lambda }&= d_\lambda ,&d_\lambda \mathbb {E}_{\lambda -\lambda _1-\lambda _2}&= 0, \\ d_\lambda \mathbb {E}_{\lambda -\lambda _2}&= - \mathbb {E}_{\lambda -\lambda _1} \mathbb {E}_{\lambda -\lambda _2} + \mathbb {E}_{\lambda -\lambda _1-\lambda _2},&d_\lambda \mathbb {E}_{\lambda -\lambda _1}&= -\mathbb {E}_{\lambda -\lambda _2} \mathbb {E}_{\lambda -\lambda _1} +\mathbb {E}_{\lambda -\lambda _1 - \lambda _2}. \end{aligned}$$

Therefore, consecutively we have

4.8 $$\begin{aligned} d_\lambda ^2&= d_\lambda + \mathbb {E}_{\lambda -\lambda _1} \mathbb {E}_{\lambda -\lambda _2} + \mathbb {E}_{\lambda - \lambda _1} \mathbb {E}_{\lambda - \lambda _1} - 2 \mathbb {E}_{\lambda -\lambda _1-\lambda _2},\\ \nonumber d_\lambda ^3&= d_\lambda ^2 - \mathbb {E}_{\lambda -\lambda _1} \mathbb {E}_{\lambda -\lambda _2} \mathbb {E}_{\lambda - \lambda _1} - \mathbb {E}_{\lambda -\lambda _2}\mathbb {E}_{\lambda -\lambda _1} \mathbb {E}_{\lambda - \lambda _2} +2\mathbb {E}_{\lambda -\lambda _1-\lambda _2},\\ \nonumber d_\lambda ^4&= d_\lambda ^3 + (\mathbb {E}_{\lambda -\lambda _1} \mathbb {E}_{\lambda - \lambda _2})^2 + (\mathbb {E}_{\lambda -\lambda _2} \mathbb {E}_{\lambda - \lambda _1})^2 - 2\mathbb {E}_{\lambda -\lambda _1-\lambda _2}. \end{aligned}$$

Hence, by Lemma 2.2,

$$\begin{aligned} d_\lambda ^4 = d_\lambda ^3 + q^{-1} \mathbb {E}_{\lambda -\lambda _1} \mathbb {E}_{\lambda - \lambda _2} + q^{-1} \mathbb {E}_{\lambda -\lambda _2} \mathbb {E}_{\lambda -\lambda _1} - 2 q^{-1} \mathbb {E}_{\lambda -\lambda _1-\lambda _2} \end{aligned}$$

which together with (4.8) implies (4.7).

Next, we consider an operator $\mathcal {T}$ defined for a function $f \in L^p(\Omega _0)$ by

$$\begin{aligned} \mathcal {T} f = \left( {d_\lambda f}: {\lambda \in P}\right) . \end{aligned}$$

We also need an operator $\widetilde{\mathcal {T}}$ acting on $g \in L^{p'}(\Omega _0)$ as

$$\begin{aligned} \widetilde{\mathcal {T}}g = \left( {-q d_\lambda ^3 g + q d_\lambda ^2 g + d_\lambda g}: {\lambda \in P}\right) . \end{aligned}$$

We observe that by two-parameter Khinchine’s inequality and Theorem 4 we have

$$\begin{aligned} \big ||\mathcal {T} f \big ||_{L^p(\ell ^2)} \lesssim {\left||f \right||}_{L^p}, \quad \text {and}\quad \big ||\widetilde{\mathcal {T}} g \big ||_{L^{p^{\prime }}(\ell ^2)} \lesssim {\left||g \right||}_{L^{p^{\prime }}}. \end{aligned}$$

The dual operator $\mathcal {T}^\star : L^{p^{\prime }}\big (\pi , \ell ^2(\mathbb {Z}^2)\big ) \rightarrow L^{p^{\prime }}(\Omega _0)$ is given by

$$\begin{aligned} \mathcal {T}^\star \vec {g} = \sum _{\lambda \in P} d_\lambda g_\lambda . \end{aligned}$$

Since $\widetilde{\mathcal {T}} g \in L^{p^{\prime }}\big (\pi , \ell ^2(\mathbb {Z}^2)\big )$, by (4.7) and Theorem 4,

$$\begin{aligned} \mathcal {T}^\star \widetilde{\mathcal {T}} g = \sum _{\lambda \in P} d_\lambda g = g \end{aligned}$$

Therefore, by Cauchy–Schwarz and Hölder inequalities

$$\begin{aligned} {\langle f, g\rangle } = {\langle f, \mathcal {T}^\star \widetilde{\mathcal {T}} g\rangle } \le \big ||\mathcal {T} f \big ||_{L^p(\ell ^2)} \big ||\widetilde{\mathcal {T}} g \big ||_{L^{p^{\prime }}(\ell ^2)} \lesssim \big ||\mathcal {T} f \big ||_{L^p(\ell ^2)} {\left||g \right||}_{L^{p^{\prime }}} \end{aligned}$$

and since ${\left||\mathcal {T}f \right||}_{L^p(\ell ^2)} = {\left||S f \right||}_{L^p}$ the proof is finished.

Finally, the method of the proof of Theorem 3, together with Theorems 4 and 5 shows the following

#### Theorem 6

Let $\left( {T_\lambda }: {\lambda \in P}\right)$ be a family of operators such that for some $\delta > 0$ and $p_0 \in (1, 2)$

$$\begin{aligned}&{{\left||{T_\lambda } \right||}_{L^{1} \rightarrow L^{1}}} \lesssim 1, \\&{{\left||{T_\mu T_\lambda ^\star } \right||}_{L^{2} \rightarrow L^{2}}} \lesssim q^{-\delta {|{\mu -\lambda } |}} \quad \text {and} \quad {{\left||{T_\mu ^\star T_\lambda } \right||}_{L^{2} \rightarrow L^{2}}} \lesssim q^{-\delta {|{\mu -\lambda } |}},\\&{{\left||{d_\lambda T_\mu d_{\lambda ^{\prime }}} \right||}_{L^{2} \rightarrow L^{2}}} \lesssim q^{-\delta {|{\lambda -\mu } |}} q^{-\delta {|{\lambda ^{\prime }-\mu } |}},\\&\big ||{\sup _{\lambda \in P} {|{T_\lambda f_\lambda } |}}\big ||_{L^{p_0}} \lesssim \big ||\sup _\lambda {|{f_\lambda } |} \big ||_{L^{p_0}}. \end{aligned}$$

Then for any $p \in (p_0, 2]$ the sum $\sum _{\lambda \in P} T_\lambda$ converges unconditionally in the strong operator topology for the operators on $L^p(\Omega _0)$.

## Appendix: About $\Omega _0$ and Heisenberg Group

In some cases $\Omega _0$ can be identified with a Heisenberg group over a nonarchimedean local field. Let us recall, that *F* is a *nonarchimedean local field* if it is a topological field^1^ that is locally compact, second countable, non-discrete and totally disconnected. Since *F* together with the additive structure is a locally compact topological group it has a Haar measure $\mu$ that is unique up to multiplicative constant. Observe that for each $x \in F$, the measure $\mu _x(B) = \mu (x B)$ is also a Haar measure. We set

$$\begin{aligned} {|{x} |} = \frac{\mu _x(B)}{\mu (B)}, \end{aligned}$$

where *B* is any measurable set with finite and positive measure. By $\mathcal {O}= \{x \in F : {|{x} |} \le 1\}$, we denote the ring of integers in *F*. We fix $\pi \in \mathfrak {p} - \mathfrak {p}^2$, where

$$\begin{aligned} \mathfrak {p} = \big \{x \in F : {|{x} |} < 1\big \}. \end{aligned}$$

We are going to sketch the construction of a building associated to ${\text {GL}}(3, F)$. For more details, we refer to [14]. A lattice is a subset $L \subset F^3$ of the form

$$\begin{aligned} L = \mathcal {O}v_1 + \mathcal {O}v_2 + \mathcal {O}v_3, \end{aligned}$$

where $\{v_1, v_2, v_3\}$ is a basis of $F^3$. We say that two lattices $L_1$ and $L_2$ are equivalent if and only if $L_1 = a L_2$ for some nonzero $a \in F$. Then $\mathscr {X}$, the building of ${\text {GL}}(3, F)$, is the set of equivalence classes of lattices in $F^3$. For $x, y \in \mathscr {X}$ there are a basis $\{v_1, v_2, v_3\}$ of $F^3$ and integers $j_1 \le j_2 \le j_3$ such that (see [14, Proposition 3.1])

$$\begin{aligned} x = \mathcal {O}v_1 + \mathcal {O}v_2 + \mathcal {O}v_3, \quad \text {and}\quad y = \pi ^{j_1} \mathcal {O}v_1 + \pi ^{j_2} \mathcal {O}v_2 + \pi ^{j_3} \mathcal {O}v_3. \end{aligned}$$

We say that *x* and *y* are joined by an edge if and only if $0 = j_1 \le j_2 \le j_3 = 1$. The subset

$$\begin{aligned} \mathscr {A} = \big \{\pi ^{j_1} \mathcal {O}v_1 + \pi ^{j_2} \mathcal {O}v_2 + \pi ^{j_3} \mathcal {O}v_3 : j_1, j_2, j_3 \in \mathbb {Z}\big \} \end{aligned}$$

is called an apartment. A sector in $\mathscr {A}$ is a subset of the form

$$\begin{aligned} \mathcal {S}= \big \{x + \pi ^{j_1} \mathcal {O}v_1 + \pi ^{j_2} \mathcal {O}v_2 + \pi ^{j_3} \mathcal {O}v_3 : j_{\sigma (1)}\le j_{\sigma (2)} \le j_{\sigma (3)}, j_1, j_2, j_3 \in \mathbb {Z}\big \}, \end{aligned}$$

where $\sigma$ is a permutation of $\{1, 2, 3\}$ and $x \in \mathscr {A}$. Thus, a subsector of $\mathcal {S}$ is

$$\begin{aligned} \big \{x + \pi ^{k_1+j_1} \mathcal {O}v_1 + \pi ^{k_2+j_2} \mathcal {O}v_2 + \pi ^{k_3+ j_3} \mathcal {O}v_3 : j_{\sigma (1)} \le j_{\sigma (2)} \le j_{\sigma (3)}, j_1, j_2, j_3 \in \mathbb {Z}\}, \end{aligned}$$

for some $0 \le k_{\sigma (1)} \le k_{\sigma (2)} \le k_{\sigma (3)}$. Finally, two sectors

$$\begin{aligned} \mathcal {S}= \big \{x + \pi ^{j_1} \mathcal {O}v_1 + \pi ^{j_2} \mathcal {O}v_2 + \pi ^{j_3} \mathcal {O}v_3 : j_{\sigma (1)}\le j_{\sigma (2)} \le j_{\sigma (3)}, j_1, j_2, j_3 \in \mathbb {Z}\big \}, \end{aligned}$$

and

$$\begin{aligned} \mathcal {S}' = \big \{x' + \pi ^{j_1} \mathcal {O}v_1 + \pi ^{j_2} \mathcal {O}v_2 + \pi ^{j_3} \mathcal {O}v_3 : j_{\sigma ^{\prime }(1)}\le j_{\sigma ^{\prime }(2)} \le j_{\sigma ^{\prime }(3)}, j_1, j_2, j_3 \in \mathbb {Z}\big \}, \end{aligned}$$

are opposite if $\sigma ^{\prime } \circ \sigma ^{-1} = (3\, 2\, 1)$.

A sector in $\mathscr {X}$ is a sector in one of its apartments. Two sectors in $\mathscr {X}$ are equivalent if and only if its intersection contains a sector. By $\Omega$, we denote the equivalence classes of sectors in $\mathscr {X}$. Let $\omega _0$ and $\omega _0^{\prime }$ be the equivalence class of

$$\begin{aligned} \mathscr {S}_0 = \big \{\pi ^{j_1} \mathcal {O}e_1 + \pi ^{j_2} \mathcal {O}e_2 + \pi ^{j_3} \mathcal {O}e_3 : j_1 \le j_2 \le j_3, j_1, j_2, j_3 \in \mathbb {Z}\big \}, \end{aligned}$$

and

$$\begin{aligned} \mathscr {S}_0^{\prime } = \big \{\pi ^{j_1} \mathcal {O}e_1 + \pi ^{j_2} \mathcal {O}e_2 + \pi ^{j_3} \mathcal {O}e_3 : j_1 \ge j_2 \ge j_3, j_1, j_2, j_3 \in \mathbb {Z}\big \}, \end{aligned}$$

respectively. Two sectors $\mathscr {S}$ and $\mathscr {S}^{\prime }$ are opposite in $\mathscr {X}$ if there are subsectors of $\mathscr {S}$ and $\mathscr {S}^{\prime }$ opposite in a common apartment. By $\Omega _0$, we denote the equivalence classes of sectors opposite to $\mathscr {S}_0$.

Suppose that $\omega ^{\prime } \in \Omega _0$. Let $\{v_1, v_2, v_3\}$ be a basis of $F^3$, and $k_1 \le k_2 \le k_3$ and $k_1^{\prime } \ge k_2^{\prime } \ge k_3^{\prime }$ be integers such that

4.9 $$\begin{aligned} \big \{\pi ^{j_1 + k_1} \mathcal {O}v_1 + \pi ^{j_2 + k_2} \mathcal {O}v_2 + \pi ^{j_3 + k_3} \mathcal {O}v_3 : j_1 \le j_2 \le j_3, j_1, j_2, j_3 \in \mathbb {Z}\big \}, \end{aligned}$$

and

4.10 $$\begin{aligned} \big \{\pi ^{j_1 + k_1^{\prime }} \mathcal {O}v_1 + \pi ^{j_2 + k_2^{\prime }} \mathcal {O}v_2 + \pi ^{j_3 + k_3^{\prime }} \mathcal {O}v_3 : j_1 \ge j_2 \ge j_3, j_1, j_2, j_3 \in \mathbb {Z}\big \}, \end{aligned}$$

belong to $\omega _0$ and $\omega ^{\prime }$, respectively. Since the sector (4.9) belongs to $\omega _0$, we have

$$\begin{aligned} v_1 = b_{11} e_1, \quad v_2 = b_{21} e_1 + b_{22} e_2, \quad v_3 = b_{31} e_1 + b_{32} e_2 + b_{33} e_3, \end{aligned}$$

for some $b_{ij} \in F$ such that $b_{11}, b_{22}, b_{33} \ne 0$. Hence, the matrix

$$\begin{aligned} g = \begin{pmatrix} b_{11} &{}\quad b_{21} &{}\quad b_{31} \\ 0 &{}\quad b_{22} &{}\quad b_{32} \\ 0 &{}\quad 0 &{}\quad b_{33} \end{pmatrix}, \end{aligned}$$

satisfies $g e_j = v_j$. In particular, $g\omega ^{\prime }_0 = \omega ^{\prime }$. Therefore, the group of upper triangular matrices acts transitively on $\Omega _0$. Observe also that the stabilizer of $\omega _0'$ in ${\text {GL}}(3, F)$ is the group of lower triangular matrices. Thus, the group

$$\begin{aligned} \left\{ \begin{pmatrix} 1 &{}\quad x &{}\quad z \\ 0 &{}\quad 1 &{}\quad y \\ 0 &{}\quad 0 &{}\quad 1 \end{pmatrix} : x, y, z \in F \right\} \end{aligned}$$

acts simply transitively on $\Omega _0$.
